# Accurate Prediction for Antibody Resistance of Clinical HIV-1 Isolates

**DOI:** 10.1038/s41598-019-50635-w

**Published:** 2019-10-11

**Authors:** Reda Rawi, Raghvendra Mall, Chen-Hsiang Shen, S. Katie Farney, Andrea Shiakolas, Jing Zhou, Halima Bensmail, Tae-Wook Chun, Nicole A. Doria-Rose, Rebecca M. Lynch, John R. Mascola, Peter D. Kwong, Gwo-Yu Chuang

**Affiliations:** 1 Vaccine Research Center, National Institute of Allergy and Infectious Diseases, National Institutes of Health, Bethesda, 20892 MD USA; 2 Qatar Computing Research Institute, Hamad Bin Khalifa University, Doha, 34110 Qatar; 3 Laboratory of Immunoregulation, National Institute of Allergy and Infectious Diseases, National Institutes of Health, Bethesda, 20892 MD USA; 4 Department of Microbiology, Immunology and Tropical Medicine, George Washington University, Washington, DC USA

**Keywords:** Machine learning, Infectious diseases

## Abstract

Broadly neutralizing antibodies (bNAbs) targeting the HIV-1 envelope glycoprotein (Env) have promising utility in prevention and treatment of HIV-1 infection, and several are currently undergoing clinical trials. Due to the high sequence diversity and mutation rate of HIV-1, viral isolates are often resistant to specific bNAbs. Currently, resistant isolates are commonly identified by time-consuming and expensive *in vitro* neutralization assays. Here, we report machine learning classifiers that accurately predict resistance of HIV-1 isolates to 33 bNAbs. Notably, our classifiers achieved an overall prediction accuracy of 96% for 212 clinical isolates from patients enrolled in four different clinical trials. Moreover, use of gradient boosting machine – a tree-based machine learning method – enabled us to identify critical features, which had high accordance with epitope residues that distinguished between antibody resistance and sensitivity. The availability of an *in silico* antibody resistance predictor should facilitate informed decisions of antibody usage and sequence-based monitoring of viral escape in clinical settings.

## Introduction

HIV-1 broadly neutralizing antibodies (bNAbs) target the envelope glycoprotein (Env) to neutralize diverse strains of HIV-1. Many such bNAbs can protect test animals from viral challenge at low bNAb concentration in sera^1–7^, supporting the use of bNAbs for HIV-1 prevention in human populations. In addition, several studies have shown that bNAbs can reduce viral load when administered during infection in both test animals^8–12^ and humans^13–16^, suggesting the utility of these bNAbs in treating HIV-infected humans.

**Figure 1:**
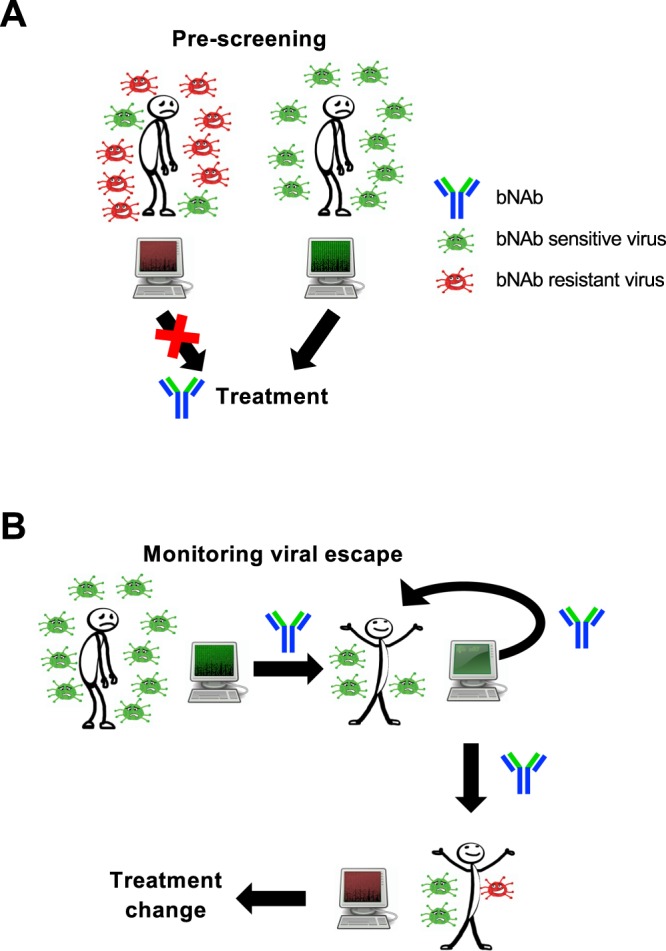
Potential clinical applications of bNAb-ReP. (**A**) bNAb-ReP can be applied during pre-screening of future patients for their neutralization susceptibility to bNAb used for treatment. (**B**) bNAb-ReP can be applied during treatment phase to monitor if viral escape to the used bNAb has occurred.

As a result of the high sequence diversity and mutation rate of HIV-1, most bNAbs cannot neutralize all HIV-1 viral isolates (Fig. 1A). Even for antibody-sensitive strains, administration of bNAbs may lead to viral escape, which can reduce or abrogate bNAb efficacy (Fig. 1A). Therefore, an efficient tool to predict HIV-1 antibody resistance could be very useful in choosing the right antibody to administer and for monitoring viral escape during the course of treatment. Furthermore, with clinical trials on several bNAbs underway, there is a pressing need for tools to analyze antibody resistance observed in these studies.

Traditionally, resistant viral strains are identified phenotypically by subcloning or synthesizing amplified Envs, producing pseudoviruses, and performing *in vitro* neutralization assay^13^, which is time consuming and expensive. In addition, resistant strains can be identified genotypically based on resistance mutations emerging in clinical samples or cell cultures under continuous antiviral pressure, for which the interpretation can be difficult as mutations may not be independent of each other^17^. Issues of interpretation are exacerbated with bNAbs which generally have complex interfaces that can tolerate considerable sequence variation.

While many genotypic assays and *in silico* algorithms have been developed to predict HIV-1 drug resistance^18^ and co-receptor usage^19^, *in silico* prediction of neutralization susceptibility to bNAbs has only been explored by a few studies^20–22^. Buiu *et al*. proposed an artificial neural network approach to model neutralization activity based on the Env sequence of an isolate. However, they assessed the performance of their predictor using only limited sequence and neutralization data of bNAb 2F5^20^. IDEPI machine learning platform predicts HIV-1 bNAb epitopes and other phenotypic features, including antibody neutralization susceptibility using sequence data^21^. The main issue with these two approaches is that they utilized only epitope residues for prediction, neglecting the influence on neutralization by regions outside the epitope. Further, both methods assumed linear relationship between amino acid diversity and neutralization sensitivity, which is most likely not given due to the high complexity of bNAb binding sites in general. More recently, Hake and Pfeifer developed support vector machine-based predictors using comprehensive sequence and neutralization data for 11 different bNAbs^22^. However, the authors did not publish their models along with the manuscript, which limits access for the general public. Magaret *et al*. developed a bNAb prediction algorithm based on Super Learner, a nonparametric ensemble-based cross-validated learning method, but only specifically for antibody VRC01^23^.

In this study, we present bNAb-Resistance Predictor (bNAb-ReP), a machine learning algorithm that predicts neutralization resistance to HIV-1 bNAbs given the sequence of the envelope, which was trained with a non-linear predictive modeling technique called gradient boosting machine (GBM). GBM trains classifiers in an additive and sequential manner by adding weak-learners one at a time while minimizing a user-defined loss function. GBM has been shown to be competitive with deep learning, particularly when large amounts of training data are not available^24,25^. Furthermore, GBM enables interpretation of the trained models by providing feature importance scores. We have generated bNAb-ReP for 33 different HIV-1 bNAbs, and these predictors can be downloaded from GitHub at https://github.com/RedaRawi/bNAb-ReP. When evaluated with neutralization data on 212 HIV-1 isolates from clinical trials, bNAb-ReP attained an overall prediction accuracy of 96%.

## Results

### bNAb-ReP training

**Figure 2:**
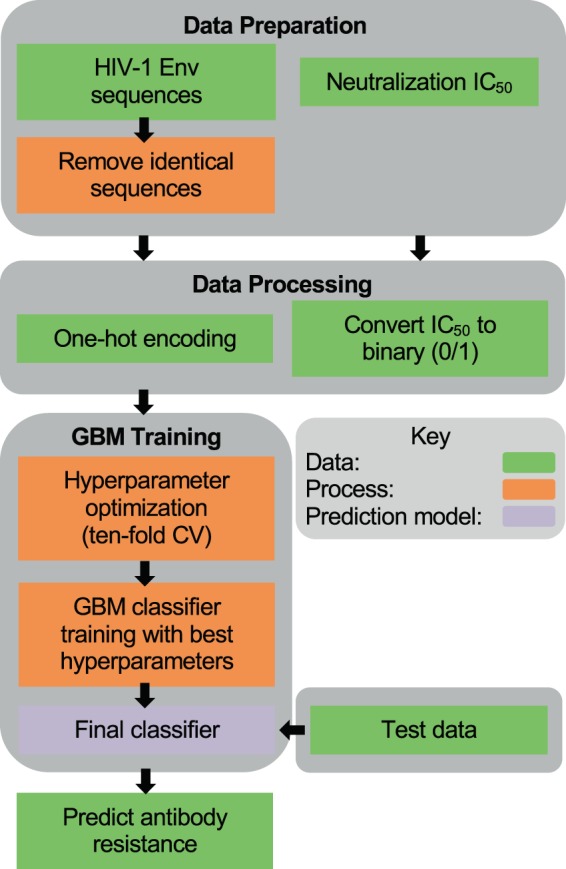
bNAb-ReP development flowchart.

**Figure 3:**
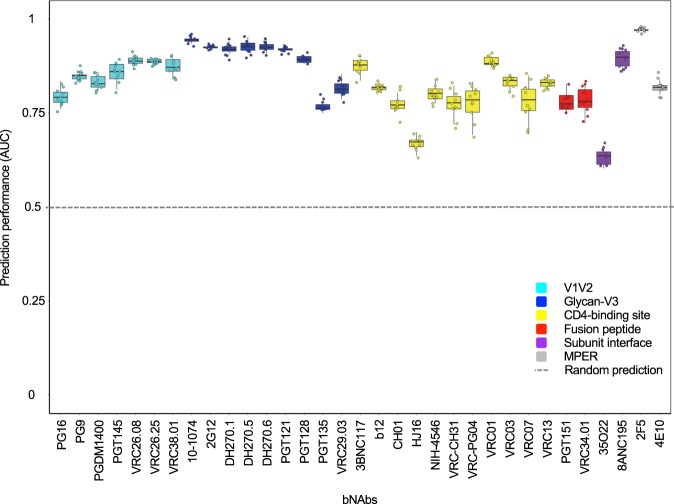
bNAb-ReP prediction performance. Prediction performance (AUC) of 33 bNAb classifiers determined by ten runs of ten-fold cross-validation, color-coded based on epitope category.

bNAb-ReP was developed using sequence and neutralization data for 33 HIV-1 bNAbs obtained from the CATNAP database^26^. The classifiers were trained using two major steps: feature generation and GBM model training (Fig. 2). In this study we used one-hot encoding of full HIV-1 Env sequences as features (see Methods), and GBM model training was performed using a hyperparameter optimization procedure to determine optimal GBM parameters for each bNAb classifier (see Methods). We evaluated the performance of all 33 bNAb-ReP GBM classifiers in ten runs of ten-fold cross-validation using previously identified optimal hyperparameters. All classifiers performed significantly better than random prediction (dashed black line in Fig. 3) with average AUC values between 0.63 and 0.97 and an overall median AUC of 0.83 (Fig. 3). The prediction performance of bNAb-ReP classifiers was also high in terms of other prediction metrics, such as accuracy, F1 score, or Matthews correlation coefficient (MCC), with average values of 0.86, 0.87, and 0.66 respectively (Supplementary Table S1). Additionally, the average root mean squared error (RMSE) was low at 0.37 (Supplementary Table S1). Notably, GBM-based classifiers showed higher AUC performance when compared to other conventional prediction methods such as logistic regression or random forest, with 22 of 33 bNAb-ReP classifiers being significantly better (Supplementary Fig. S1 and Table S1). Additionally, GBM-based classifiers showed lower RMSE when compared to logistic regression or random forest, with 28 of 33 bNAb-ReP classifiers having significantly lower error rates (Supplementary Table S2).

### bNAb-ReP feature importance

**Figure 4:**
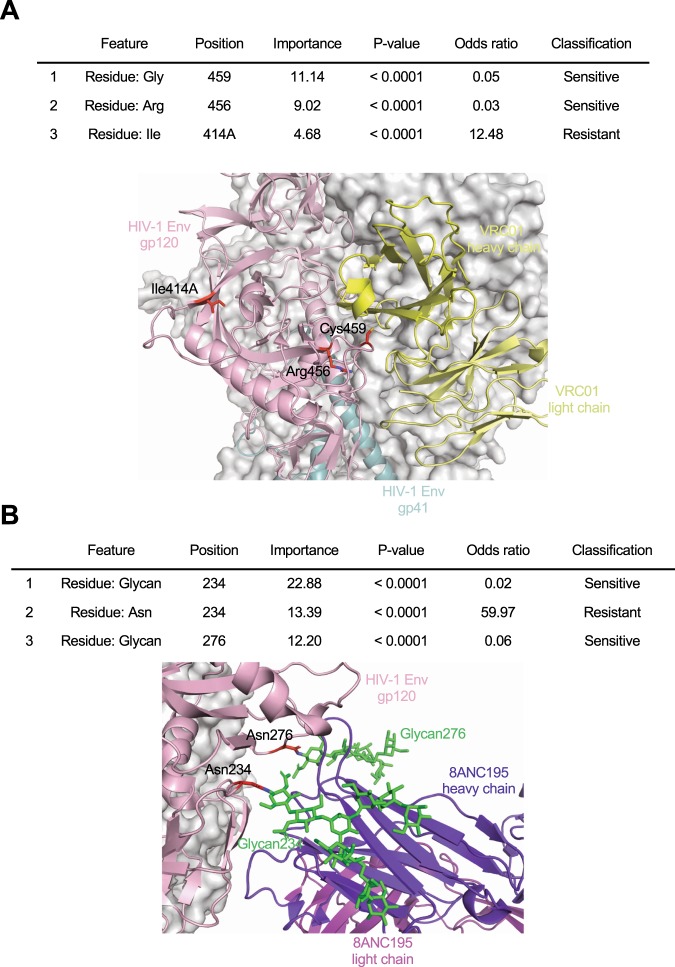
Top three discriminative features for VRC01 and 8ANC195 classifier. (**A**) The top three discriminant features of the bNAb VRC01 classifier are listed in the table and highlighted on the prefusion-closed Env trimer structure in complex with VRC01 antibody (PDB ID: 5FYJ). (**B**) The top three discriminant features of the bNAb 8ANC195 classifier are listed in the table and highlighted in the Env trimer structure in complex with 8ANC195 bNAb, with glycans 234 and 276 depicted as green sticks (PDB ID: 5CJX).

In contrast to other standard machine learning approaches such as neural networks and support vector machine, the major advantage of the tree-based methods, such as GBM, is the ability to obtain feature importance scores, the increase in the prediction error of the model after the feature’s values are permutated for all input features, which enables interpretability of the predictive models (Supplementary Table S3). For instance, the top three discriminative features of the bNAb-ReP VRC01 classifier involved HIV-1 Env residues 414A, 456, and 459 with a total feature importance of 24.84% (Fig. 4A, Supplementary Table S3). Structural studies revealed two of the three amino acid positions were located at the VRC01 epitope and thus can be critical to VRC01 binding and neutralization (Fig. 4A)^27,28^. Additionally, the top three features of bNAb-ReP 8ANC195 classifier accounted for a total variable importance of 48.47% and included Env residues 234 and 276, which must be glycosylated in order for 8ANC195 to bind and neutralize Env (Fig. 4B, Supplementary Table S3)^29^. For 21 of the 33 bNAbs for which the structural epitopes have been defined, 68% of the features with importance greater than 5% were associated with structural epitope residues (Table 1), while 32% of the features were associated regions distant from the structural epitope, suggesting that neutralization susceptibility of HIV-1 strains is not exclusively determined by epitope residues. Several epitope-distant features were associated with *N*-linked glycosylation sequons (e.g. 334_@ and 334_S for antibodies 10–1074 and PGT128, 334_S for antibody PGT135). To investigate this further, we trained predictors using only the structural epitope residues and compared to the predictors that utilized full HIV-1 Env sequences. In 18 out of 33 cases the training accuracy was significantly higher when predictors were trained using full Env sequences, while for 9 out of 33 cases the training accuracy was significantly higher when predictors were trained using only the structural epitopes (Supplementary Fig. S2). In particular, the prediction accuracy for glycan-V3 targeting bNAbs had the highest decrease when using only structural epitope residues rather than full Env sequences.

**Table 1:** Features with variable importance of greater than 5% for 21 bNAb-ReP predictors.

bNAb	Features with variable importance of greater than 5%
10–1074	**332_@**, 334_@, 334_S
2F5	**667_A**, **665_K**, 492_K, **665_S**
2G12	**295_@**, **332_@**, 395_W
3BNC117	**456_R**, **459_G**, 723_S, 466_E
4E10	787B_L, **674_D**
8ANC195	**234_@**, **234_N**, **276_@**, 349_L
b12	185_D
HJ16	**471_G**
NIH45–46	364_S, **456_R**, **279_D**
PG16	**160_@**
PG9	**160_@**, **169_E**
PGT128	334_S, **332_@**, 334_@
PGT135	334_S, **332_@**, 179_L, 592_L
PGT145	**160_@**, **169_E**
PGT151	651_N, 602_L, **519_I**, **514_G**, 629_L
VRC-CH31	**276_D**, **459_G**
VRC-PG04	**364_H**, **276_@**, **459_G**, **365_S**, **456_R**, **429_G**, **389_S**
VRC01	**459_G**, **456_R**
VRC13	**471_E**, **471_G**, 179_L
VRC34.01	**518_V**
VRC38.01	**130_@**, **171_K**

Features that were associated with epitope residues are highlighted in bold. @ denotes N-linked glycan sequon.

### Antibody resistance prediction for clinical HIV-1 isolates

**Figure 5:**
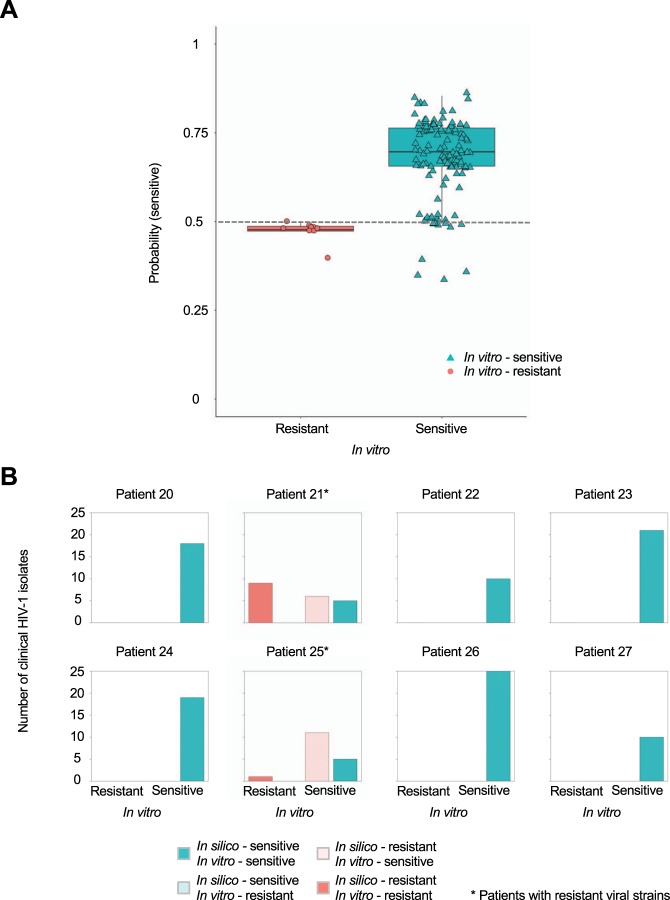
bNAb-ReP prediction performance on VRC601 clinical HIV-1 isolates. (**A**) Prediction performance of the susceptibility of VRC601 clinical isolates to VRC01. *In vitro* assay neutralization classification is shown on the x-axis, with the *in silico* predicted probability for a sequence to be sensitive to VRC01 shown on the y-axis. The classification cutoff of 0.5 is depicted with a grey dashed line. (**B**) Bar plots depicting the number of *in vitro* classified VRC601 HIV-1 isolates per patient. Clinical HIV-1 isolates *in silico* predictions are shown in red (resistant) and cyan (sensitive) with darker colors indicating true predictions and light colors indicating false predictions.

To validate bNAb-ReP beyond the datasets obtained from CATNAP, we predicted antibody resistance of HIV-1 isolates from clinical studies with HIV-1 infected individuals based on Env sequences. First, we tested the bNAb-ReP classifier for antibody VRC01 on HIV-1 isolates obtained from HIV-1 positive patients enrolled in the VRC601 trial^13^, which investigated the efficacy of VRC01 as a therapeutic to control viral load. bNAb-ReP correctly predicted 100% of the VRC01-resistant strains and 87% of the VRC01-sensitive strains (Fig. 5A). Notably, sensitive strains that were incorrectly predicted as resistant strains were all isolated from patients that contained resistant strains (Fig. 5B).

Additionally, we evaluated the prediction performance of bNAb-ReP by using sequence and neutralization data from a phase IIa clinical trial studying HIV-1 positive patients treated with bNAb 3BNC117^14^. The overall classification accuracy of bNAb-ReP was 87%, correctly predicting 26 of 29 sensitive HIV-1 Env strains, although falsely predicting the only resistant strain as sensitive (Supplementary Fig. S3).

**Figure 6:**
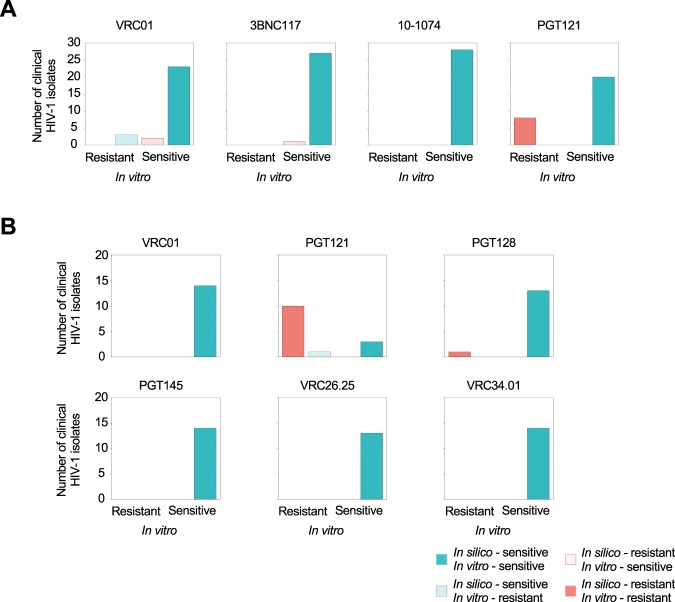
bNAb-ReP prediction performance on clinical HIV-1 isolates from Bar *et al*. and Ssemwanga *et al*. studies. (**A**) Bar plots highlighting the number of clinical HIV-1 isolates, introduced in the Bar *et al*. study, separated according to their *in silico* predictions. Resistant *in silico* predictions for bNAbs VRC01, 3BNC117, 10–1074, and PGT121 are shown in red and sensitive in cyan, with darker colors representing accurate predictions and light colors inaccurate ones, respectively. (**B**) Bar plots depicting the number of isolates, introduced by Ssemwanga *et al*., with resistant *in silico* predictions shown in red and sensitive in cyan.

To further evaluate bNAb-ReP’s prediction accuracy, we performed *in vitro* neutralization assay experiments on clinical sequences obtained from HIV-infected individuals who were undergoing analytic treatment interruption (VRC01-ATI) (Supplementary Data S1 and Table S4)^30^. bNAb-ReP predicted neutralization susceptibility to VRC01, 3BNC117, 10–1074, and PGT121 with accuracies of 82%, 96%, 100%, and 100%, respectively (Fig. 6A).

In addition to predicting neutralization susceptibility from the aforementioned clade B sequences, we used bNAb-ReP to predict resistance for clade A and A/D recombinant sequences from a superinfection case study in a Ugandan couple^31^. Compellingly, the bNAb-ReP classification accuracy achieved 100%, 93%, 100%, 100%, 100%, and 100%, for bNAbs VRC01, PGT121, PGT128, PGT145, VRC26.25, and VRC34.01, respectively (Fig. 6B).

Finally, we implemented the support vector machine (SVM) algorithm developed by Hake and Pfeifer for predicting antibody resistance and compared the performances using several independent test sets (Supplementary Table S5)^22^. bNAb-ReP outperformed Hake and Pfeifer’s SVM approach on all independent bNAb test sets, except the 3BNC117 test set.

## Discussion

The development of *in silico* sequence-based bNAb neutralization resistance prediction tools with high accuracy continues to be highly desired. In this study, we developed bNAb-ReP, a neutralization resistance predictor, for 33 bNAbs using the machine learning technique GBM. bNAb-ReP yielded an overall accuracy of 96% in predicting neutralization resistance of 212 HIV-1 sequences isolated from patients enrolled in four different clinical trials. Feature importance analyses of the predictors showed that although most of the features with high importance were associated with epitope residues, a substantial amount of features associated with residue positions distant from the epitope, exemplifying the complexity of predicting neutralization resistance. These predictors should have high utility in choosing the right antibody treatment for HIV-1 infected patients and in monitoring the development of resistance strains while on treatment. We have deposited these predictors on GitHub for public use.

We have shown that the bNAb-ReP predictors performed better than a number of different algorithms, including random forest, logistics regression, and the SVM algorithm proposed by Hake and Pfeifer^22^. Notably, we achieved high prediction accuracy using only one-hot encoding of residue types for each Env position as input features. Inclusion of other features, such as amino acid properties or additional structural features, could potentially further enhance the performance of bNAb-ReP and similar prediction approaches. It is worth noting that a bNAb resistance predictor based on combination of molecular modeling and machine learning was recently published^32^. In addition to being more time consuming for both training and testing, the analysis assumed the antigen to be conserved over the sequence space, which as the authors pointed out may not always be true, especially for regions with multiple insertions and deletions, as is the case with variable loops on HIV-1 Env.

One major limitation of machine learning models to predict bNAb resistance is the availability of the training dataset. Although hundreds of sequences with neutralization data were available for dozens of bNAbs, these inputs were still sparse given that the length of HIV-1 Env sequences is more than eight hundred residues. Thus, deep learning algorithms may not have advantages over other algorithms in this case. In addition, the limited coverage of sequence space by the training set can also give high feature importance for a few select features, while neglecting other important features that cannot be captured from the training set. Furthermore, since the training requires at least a certain amount of both sensitive and resistant sequences as input, it is not applicable to antibodies, such as 10E8 and N6, with close to 100% neutralization breadth based on strains that have been tested so far. Finally, we obtained sequence/neutralization data against eight different antibodies from clinical studies as test sets to validate our predictors objectively, but for six of these antibodies there was no more than one resistant sequence. Thus, it was not feasible to use metrics such as AUC to evaluate the performance of the predictors in addition to prediction accuracy.

As there are diverse variables impacting neutralization measurements, it was inevitable that there would be a certain degree of noise in the training data. We showed that in the case for antibody VRC01, our bNAb-ReP algorithm can tolerate a certain degree of noise in the training set (Supplementary Fig. S4). Further investigation on the impact of noisy data in the training set would be required to generalize the impact of noise to prediction performance. In addition, we have identified a number of features with high variable importance that were distant from the epitopes. Further investigation would be required to understand the influence of these features on the sensitivity and resistance of the corresponding antibodies.

## Methods

### Training data

We used the neutralization data of 33 different antibodies (10–1074, 2F5, 2G12, 35O22, 3BNC117, 4E10, 8ANC195, CH01, DH270.1, DH270.5, DH270.6, HJ16, NIH-4546, PG16, PG9, PGDM1400, PGT121, PGT128, PGT135, PGT145, PGT151, VRC-CH31, VRC-PG04, VRC01, VRC03, VRC07, VRC13, VRC26.08, VRC26.25, VRC29.03, VRC34.01, VRC38.01, and b12) assayed respectively against 205 to 711 HIV-1 isolates published in the CATNAP database^26^ as the training set. Clade distribution of the Env sequences in the training set is shown in Supplementary Fig. S5. The neutralization assays were performed using single-round-of-infection Env-pseudoviruses on cell lines^33,34^. Each HIV-1 isolate is represented with its full-length envelope glycoprotein amino acid sequence. Duplicated full-length HIV-1 envelope sequences were removed. The viral isolate was categorized as resistant to an antibody if its geometric mean IC_50_ is greater than 50 μg/ml or designated with a “>” sign, otherwise it was categorized as sensitive.

### Test data

For VRC601 clinical trial data, we used sequences and neutralization data from Env-pseudoviruses generated from Envs isolated by single genome amplification (SGA) RT-PCR from plasma virus, as described in Lynch *et al*.^13^. The Env-pseudoviruses were assayed on TZM-bl cells as in Sarzotti-Kelsoe *et al*.^34^. The clade A and A/D sequences and neutralization data were generated the same way and were taken from Ssemwanga *et al*.^31^. The VRC01-ATI sequences were derived from patients in an analytical treatment interruption trial in which volunteers based at the NIH were administered VRC01 infusions before and during an interruption of antiretroviral therapy^30^. In that publication, Env sequences were generated by SGA; however, the published neutralization assays were performed with infectious virus from outgrowth cultures, not in the Env-pseudovirus/TZM-bl format. Here, we report new data, for which we expressed the Env-pseudoviruses from the sequences reported in Bar *et al*. and used the TZM-bl format as above^30^.

### Gradient boosting machine

To build the training models, we employed a non-linear interpretable tree-based ensemble technique referred to as a gradient boosting machine (GBM) for building antibody resistance predictors using *h2o* package (Version 3.16.0.2) in *R* software (https://www.R-project.org)^35,36^. GBM belongs to the family of predictive methods which uses an iterative strategy such that the learning framework will consecutively fit new models to have a more accurate estimate of the response variable after each iteration. The primary notion behind this technique is to construct new tree-based learners to be as correlated as possible with the negative gradient of a given loss function, calculated using all the training data. We can use any arbitrary loss function (L(·,·)) here. However, if the loss function is the most commonly used squared-loss function, the learning procedure would result in consecutive residual error-fitting. Algorithm 1 summarizes the generic GBM approach.

**Algorithm 1:**
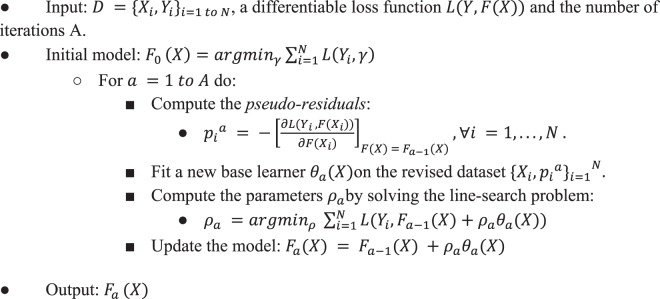
Gradient Boosting Machine.

The advantage of the boosting procedure is that it works on decreasing the bias of the model, without increasing the variance. Learning uncorrelated base learners helps to reduce the bias of the final ensemble model. In this work, we used the *L*_2_- TreeBoost approach proposed by Friedman^35^ to build the core GBM model. Here the loss function is the classical squared-loss function (*L*_2_):

$${L}_{2}=\frac{1}{2}||Y-F(X)|{|}_{2}^{2},Y\in \{0,1\}.$$

In our approach, the base learner is a *J*-terminal node classification tree. Each tree model has an additive form given as:

$$\theta {(X;\{{\gamma }_{j},{P}_{j}\})}_{j=1}^{J}=\mathop{\sum }\limits_{j=1}^{J}\,{\gamma }_{j}1(X\in {P}_{j}).$$

here ${\{{P}_{j}\}}_{1}^{j}$ are J disjoint regions that together cover the space of all joint values of the predictor variable *X*. These regions represent the J terminal nodes of the corresponding classification tree. The indicator function 1(·) takes the value 1 if the argument passed to it is true, and 0 otherwise. Because the regions are disjoint, *θ*(*X*) is equivalent to the prediction rule: *if X* ∈ *P*_*j*_, *then θ*(*X*) = *γ*_*j*_. Now, the pseudo-residuals become:

$${p}_{i}^{a}=-{[\frac{\partial {L}_{2}({Y}_{i},F({X}_{i}))}{\partial F({X}_{i})}]}_{F(X)={F}_{a-1}(X)}={Y}_{i}-{F}_{a-1}({X}_{i}),\forall i=1,\mathrm{...}\,,N$$

The line search becomes:

$$\begin{array}{rcl}{\rho }_{a} & = & argmi{n}_{\rho }\mathop{\sum }\limits_{i=1}^{N}||{Y}_{i}-{F}_{a-1}({X}_{i})-{\rho }_{a}{\theta }_{a}({X}_{i})|{|}_{2}^{2}\\  & = & argmi{n}_{\rho }\mathop{\sum }\limits_{i=1}^{N}||{p}_{i}^{a}-{\rho }_{a}{\theta }_{a}({X}_{i})|{|}_{2}^{2}\end{array}$$

Using classification trees as base learners, we use the idea of separate updates for each terminal region *P*_*j*_^*a*^ as proposed in^35^ to get:

1 $${\rho }_{j}^{a\,}=mea{n}_{{X}_{i}\in {P}_{j}^{a}}({\gamma }_{j}^{a}{p}_{i}^{a})$$

The *L*_2_- TreeBoost approach for two-class GBM is summarized in Algorithm 2.

**Algorithm 2:**
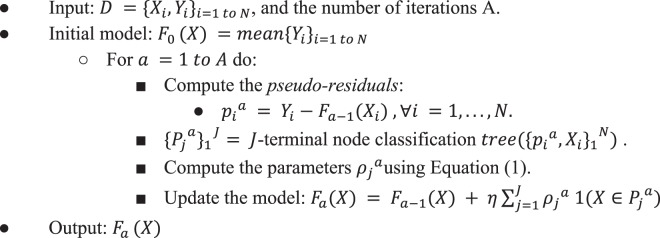
*L*_2_- TreeBoost method for GBM.

Here the parameter *η* is a regularization parameter which is used to avoid overfitting the models and is acquired via cross validation. For each iteration a, the least-squares criterion (*I*(*ϕ*)) used to assess potential splits of a current terminal region *P* into two disjoint sub-regions (*P*_*l*_, *P*_*r*_) is given by:

2 $${i}^{2}({P}_{l},{P}_{r})=I(\varphi )=\frac{{w}_{l}{w}_{r}}{{w}_{l}+{w}_{r}}{({Y}_{l}-{Y}_{r})}^{2},$$

where *Y*_*l*_ and *Y*_*r*_ are the left and right child node responses respectively, and *w*_*l*_,*w*_*r*_ are proportional to the number of samples in regions *P*_*l*_ and *P*_*r*_ respectively as show in (Friedman^35^). *I*(*ϕ*) is a measure of the importance of the variable (*ϕ*) which maximizes this criterion. During a given iteration, only one feature is allowed to cause a split into two terminal regions. Thus, in the case of a *J*-terminal node classification tree, we generate *J* − 1 such measures. However, the same feature can generate multiple optimal splits for the *J*-terminal node tree. In such a scenario, we sum the importance of such features to get the total importance of each feature *ϕ* after A iterations. This procedure results in the variable importance scores from the GBM approach.

### Classifier features

Sequence information was represented using one-hot encoding to represent 20 standard amino acids and N-linked glycan. Each amino acid aa_i_, ${\rm{i}}\,\epsilon \,\{1,\ldots ,21\}$ was translated into a 21-dimensional vector, where the i^th^ vector position was set to 1, and all other 20 vector positions were set to 0. For instance, applying one-hot encoding to an amino acid sequence of length 100, would be translated into a binary vector of length 2100.

### Training of bNAb-ReP

To train bNAb-ReP classifiers, we first performed a hyperparameter optimization to identify the optimal GBM parameters for the given data. We created a grid of *T* × *J* × *r* × *η* = 120, in particular number of trees **T** = 1000, maximum depth ${\rm{J}}\,{\boldsymbol{\epsilon }}\,\{1,2,3,4,5,6\}$, sample rate ${\bf{r}}\,{\boldsymbol{\epsilon }}\,\{\frac{\sqrt{\#{\bf{f}}{\bf{e}}{\bf{a}}{\bf{t}}{\bf{u}}{\bf{r}}{\bf{e}}{\bf{s}}}}{\#{\bf{f}}{\bf{e}}{\bf{a}}{\bf{t}}{\bf{u}}{\bf{r}}{\bf{e}}{\bf{s}}},\,0.1,\,0.2,\,0.3\}$, and learn rate ${\rm{\eta }}\,{\boldsymbol{\epsilon }}\,\{0.001,0.01,0.05,0.1,0.2\}$. Worth noting is that we apply an early stopping criterion based on convergence of the training area-under-the-curve (AUC). This has a particular effect on parameter ***T***, which only in 2 out of 33 cases exceeds the value 100. Subsequently, we performed for each combination ten-fold cross-validation and selected the parameters that yielded the maximal ten-fold cross validation AUC values. We then performed ten-fold cross validation for each of the combinations. Finally, we selected the best parameters that had the maximal ten-fold cross validation area under the curve (AUC). Once the optimal hyper-parameters are known, the models were built on the full training set using these parameters and their prediction performance were evaluated on the independent test sets.

### Alternative predictors

For comparison to the bNAb-ReP predictors, we trained additional models based on logistic regression and random forest. Logistic Regression belongs to the class of generalized linear models and we trained binomial predictors using *glm* function available in *h2o* package in *R*. Random Forest (RF) belongs to the class of ensemble-based supervised tree-based learning techniques. The RF algorithm applies the general technique of bagging or bootstrapped aggregating to decision tree learners. We performed a grid search for optimizing the hyper-parameters including the number of trees in the random forest, maximum depth of the trees and column sampling rate using a ten-fold cross-validation strategy. We used the distributed random forest function for implementing random forest models, available in *h2o* package in *R*. Further, we implemented the support vector machines (SVM) algorithm proposed by Hake and Pfeifer^22^. Support vector machines (SVM) belong to the family of non-linear optimization technique used to distinguish input data associated with different classes by constructing separating hyperplanes. A crucial step in building SVM models is the choice of the non-linear kernel function that encodes the similarity structure in the input data. The kernel function takes the input data to a high dimensional space where the inputs belonging to each class are linearly separable, which when mapped back to the input space results in non-linear separating hyperplanes. In this work, we used the oligo kernel as proposed by Hake and Pfeifer for all the bNAbs to predict the neutralization susceptibility to each bNAb for new viral strains^22^, using the exact implementation and training data proposed by Hake and Pfeifer^22^ (script is available under: https://github.com/RedaRawi/bNAb-ReP).

All model training scripts are available under: https://github.com/RedaRawi/bNAb-ReP.

### Derivation of probability threshold to categorize sensitivity and resistance

Though there is no clear relationship between the proportion of training/testing split and the model performance, Shabin *et al*. identified that the best results were obtained when 75% of the whole dataset was used for training and 25% for testing^37^. Similar as implemented by Pfeifer *et al*. and Hake *et al*., we used that probability cutoff as the optimal threshold to distinguish between resistant and sensitive viral sequences^22,38^. In particular, we chose for each bNAb classifier a cutoff that provided the best balance between average true positive and true negative rate.

### Noise Simulation

We examined how random noise will affect the prediction performance of bNAb-ReP predictor for antibody VRC01. We trained bNAb-ReP predictor using the original 640 training sequences/neutralization categories with 320, 128, 64, 43, 32, 21, and 13 noise sequences/neutralization categories added respectively (equivalent to a signal to noise ratio (SNR) of 2, 5, 10, 15, 20, 30, 50, respectively). For each random noise input, the amino acid for each residue position of the noise sequence and neutralization category is randomly sampled based on their prevalence in the original VRC01 training set. Each SNR experiment was repeated five times.

### Epitope and paratope buried surface area calculations

The buried surface area between antibody and antigen was calculated using NACCESS software^39,40^. The epitope and paratope residues for each antibody were defined as residues with non-zero buried surface area. In the case of 2G12, the epitope residues were defined as glycans N295, N332, N339, N386, and N392, based on Scanlan *et al*.^41^. The final epitope residues for each category were defined as follows. V1V2 category epitope residues comprised all alignment positions between residue numbers 131–196 (HXB2 numbering). The epitope residues for all other categories were defined as the union of all bNAb epitope residues within each category determined as described above.

### Statistical analyses

P-values and odds ratio values presented in Fig. 4A,B were calculated using Fisher’s exact test (*R* function *fisher*.*test*). Statistical significance, presented in Supplementary Figs S1 and S2, was determined using the following procedure. First, we tested the list of AUC values for normal distribution using *R* library *nortest*, in particular function *ad*.*test*. If normal distribution and additionally variance homogeneity were given (*R* function *var*.*test*), we used t-test to determine significance (*R* function *t*.*test*). If neither normal distribution nor variance homogeneity were given, we applied Mann-Whitney test (*R* function *wilcox*.*test*).

## Supplementary information

Supplementary Info

Supplementary Table S1

Supplementary Table S2

Supplementary Table S3

Supplementary Table S4

Supplementary Table S5

## Data Availability

We provide all neutralization and sequence data, the resistance predictors for 33 broadly neutralizing HIV-1 antibodies, as well as scripts to build new bNAb-ReP predictors at https://github.com/RedaRawi/bNAb-ReP.
