# Supplementary material for: Accurate Prediction for Antibody Resistance of Clinical HIV-1 Isolates
Source: Sci Rep. 2019 Oct 11;9:14696. doi: 10.1038/s41598-019-50635-w (PMC6789020; doi:10.1038/s41598-019-50635-w)
Supplement: Supplementary file 1 — Supplementary Info [file 41598_2019_50635_MOESM1_ESM.pdf]

Accurate Prediction for Antibody Resistance of Clinical HIV-1 Isolates

Reda Rawi[1], Raghvendra Mall[2], Chen-Hsiang Shen[1], S. Katie Farney[1], Andrea Shiakolas[1], Jing Zhou[1], Halima Bensmail[2], Tae-Wook Chun[3], Nicole A. Doria-Rose[1], Rebecca M. Lynch[4], John R. Mascola[1], Peter D. Kwong[1] & Gwo-Yu Chuang[1*]

[1]*Vaccine Research Center, National Institute of Allergy and Infectious Diseases, National Institutes of Health, Bethesda, 20892, MD, USA*

[2]*Qatar Computing Research Institute, Hamad Bin Khalifa University, Doha, 34110, Qatar*

[3]*Laboratory of Immunoregulation, National Institute of Allergy and Infectious Diseases, National Institutes of Health, Bethesda, 20892, MD, USA*

[4]*Department of Microbiology, Immunology and Tropical Medicine, George Washington University, Washington, DC, USA*

* Corresponding author: gwo-yu.chuang@nih.gov

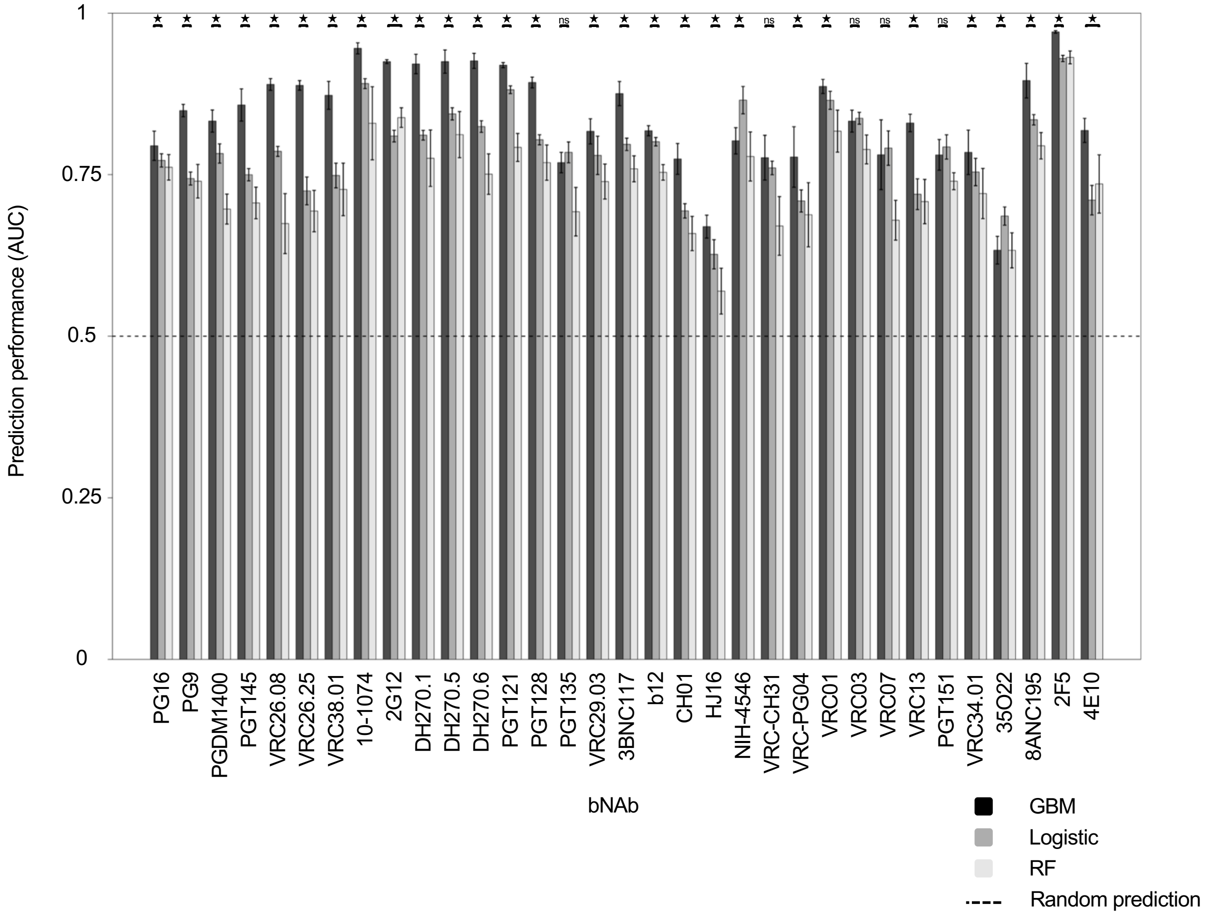

Figure S1. Prediction performance of GBM classifiers compared to Logistic Regression and Random Forest predictors.

Prediction performance (AUC) comparison of GBM (black bars), logistic regression (dark gray), and random forest (light gray) of 33 bNAb prediction models determined by ten runs of ten-fold cross-validation. For the sake of clarity, results from statistical significance testing are shown only for the top 2 models (*P-value < 0.05, ns = not significant).

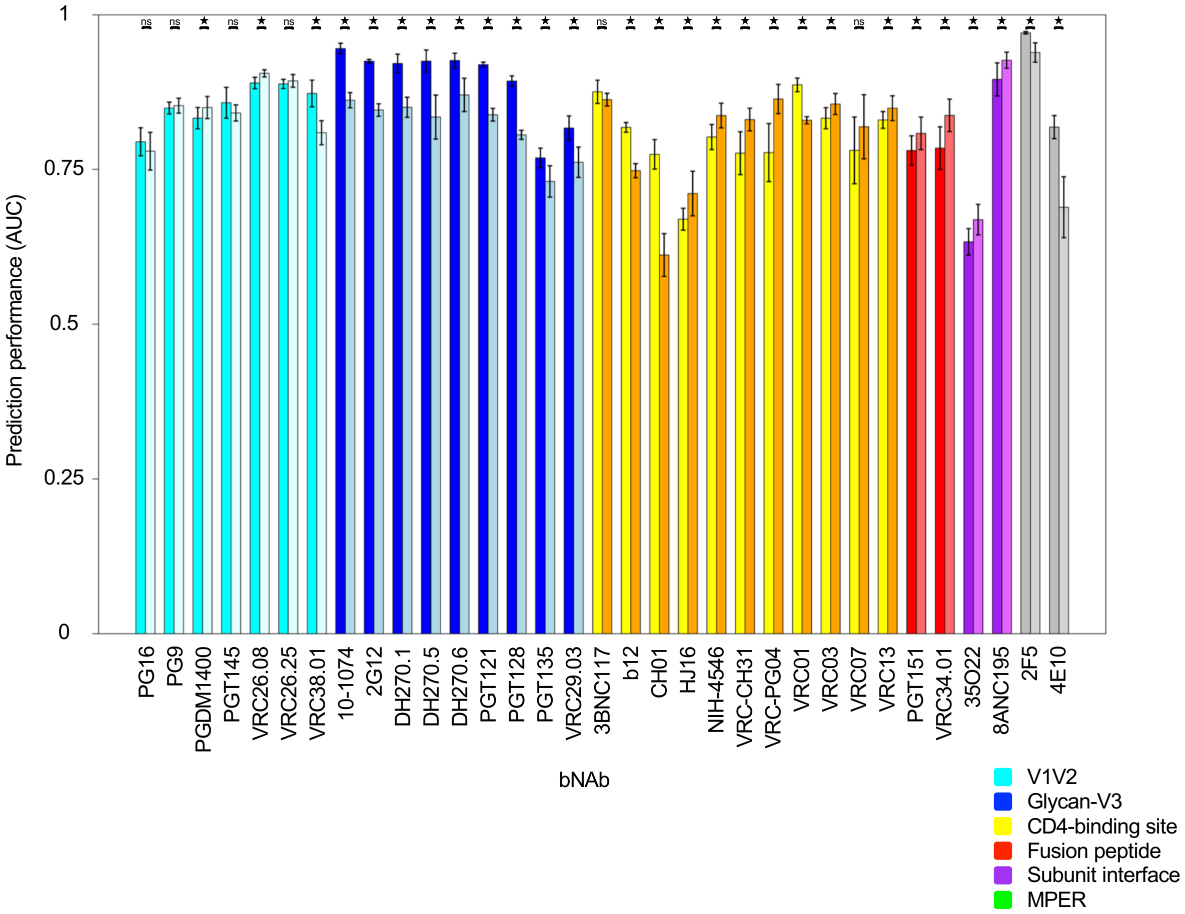

Figure S2. Prediction performance of bNAb classifiers using sequences comprising the full Env sequences or the epitope region only.

The prediction performance of the bNAb classifiers using full Env sequences or sequence subsets comprising epitope region only were determined by ten runs of ten-fold cross-validation and are shown as bar plots. The 33 different classifiers are shown on the x-axis, named according to the antibody they are trained on. The colors of the bars refer to the epitope category of the corresponding antibody. The left bar of each pair of bars refers to classifiers trained using full Env sequences, while the right bar refers to classifiers trained using epitope sequence subsets only.

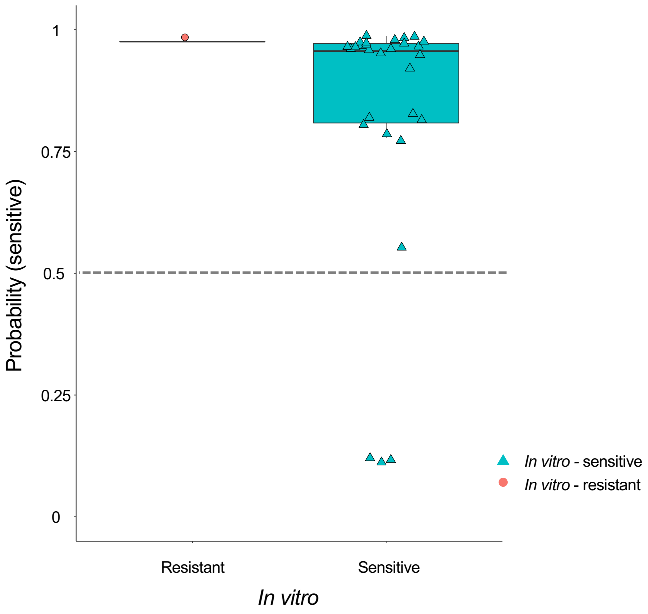

Figure S3. Prediction performance of bNAb-ReP on Env strains from bNAb 3BNC117 treatment study.

Prediction performance on bNAb 3BNC117 neutralization and sequence data shown as boxplot. *In vitro* assay neutralization classification is shown on the x-axis, with the *in silico* predicted probability for a sequence to be sensitive to 3BNC117 shown on the y-axis. The classification cutoff of 0.5 is depicted with a grey dashed line.

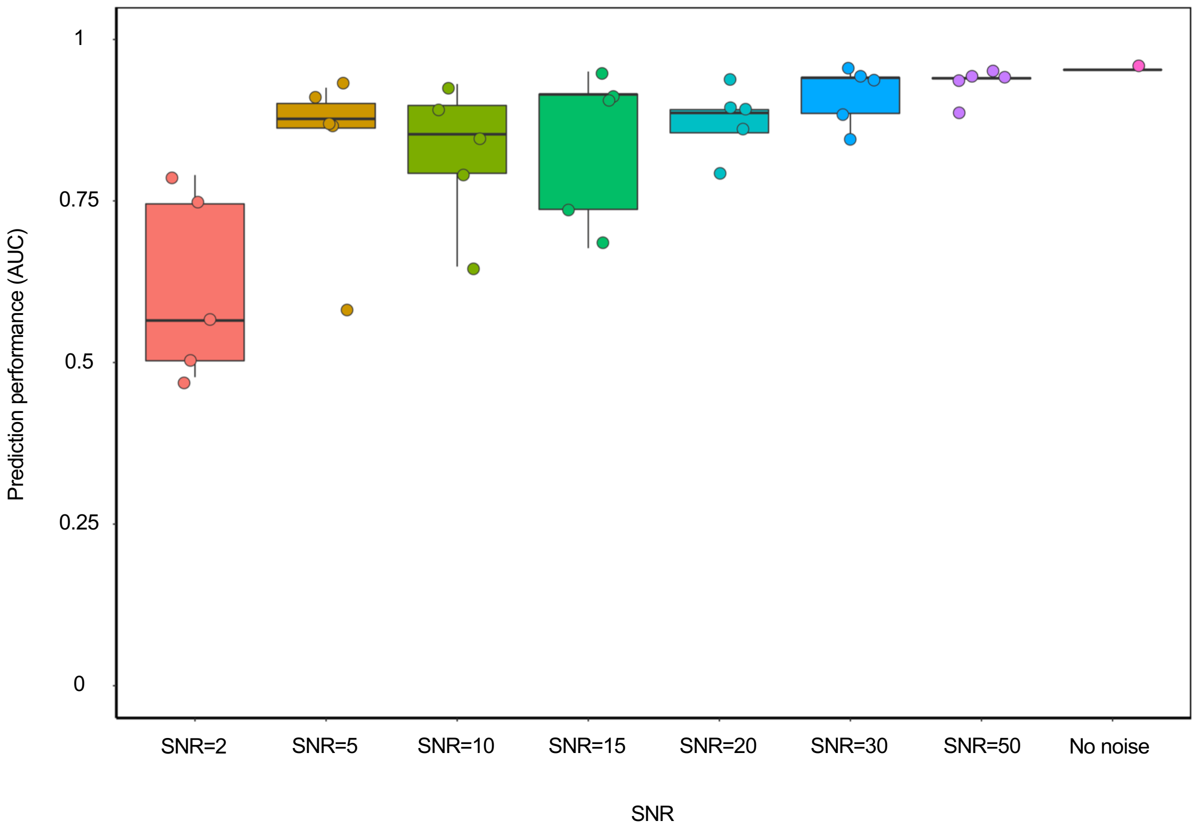

Figure S4. Prediction performance of VRC01 bNAb-ReP with noise added to training set. The prediction performance (AUC) of VRC01 bNAb-ReP with signal to noise ratio (SNR) = 2, 5, 10, 15, 20, 30, 50 on the Lynch et al. VRC01 test set is shown. Each SNR experiment was repeated five times.

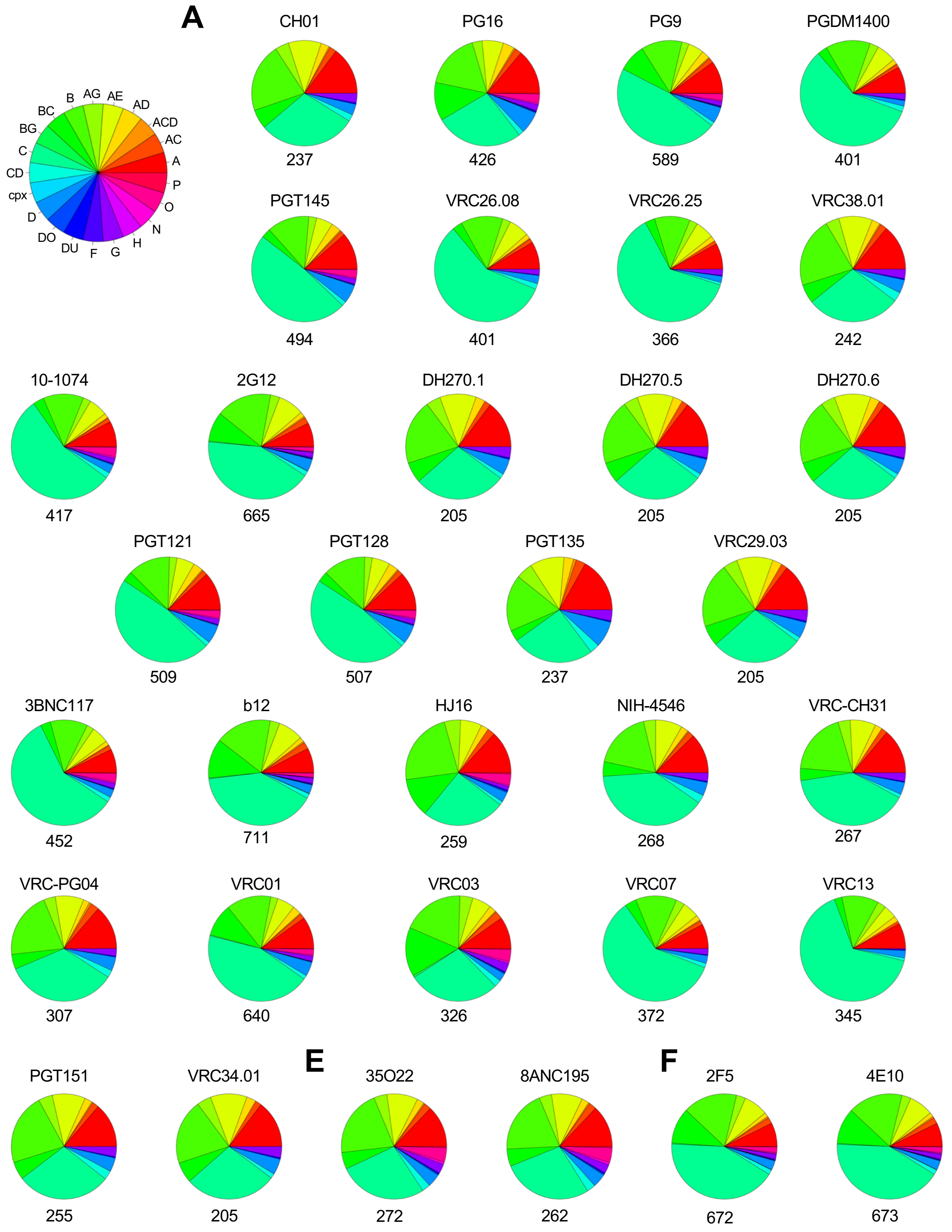

Figure S5. Training sets clade distributions

Clade distributions for each bNAb training set are illustrated as pie charts, with distinct epitope categories shown in (**A**) V1V2, (**B**) Glycan-V3, (**C**) CD4-binding site, (**D**) fusion peptide, (**E**) subunit interface, and (**F**) MPER. The number of strains within each training sets is depicted below the corresponding pie chart.

Supplementary Data S1. HIV-1 Env sequences of VRC01-ATI study

>V08DA_POVIp_2H3.A.9
MRVRGIRKNYQRLWKWG-TLLSMLLGLLMICNAKEQLWVTVYYGVPVWKEANTTLFCASD
ARAYSTEKHNVWATHACVPTDPDPQEVKLGNVTENFNMWKNNMVDQMHEDIISLWDQSLK
PCVEITPLCVTLNCTDYKGNASSTN------NSGGEMENKEEMKNCSFNITTSLSDKV-Q
ERALFYIYDITPINN-----TTNTSYKLTRCNSSVITQACPKVSFEPIPIHYCAPAGFAI
LKCRQKNFKGTGPCTDVSTVQCTHGIRPVVSTQLLLNGSLAEDEVVIKSENLTDNSKNII
VQLQEAINITCTRPNNNTRKSINI--GPGRAIYATGEIIGDIRQAHCNISEEKWNKTLGQ
IVEKLRKQFN-NKTIVFAQPSGGDPEIVMHSFNCGGEFFYCNTTRLFNSTWSTWNDS-ST
LSNGTGNNTITLPCRIKQVINMWQKVGKAMYAPPIAGQIRCSSNITGLILTRDGG----S
NNMSNETFRPGGGNMKDNWRSELYKYKVVKIEPLGIAPTKAKRRVVQREKRAIG-IGAVF
LGFLGAAGSTMGAASVTLTVQARQLLFGIVQQQNNLLRAIEAQQHLLQLTVWGIKQLQAR
VLAVERYLKDQQLLGIWGCSGKLICTTAVPWNTSWSNKSLEQIWDNMTWMQWEKEINNYT
GLIYTLIEQSQNQQEKNEQELLELDTWASLWNWFDISKWLWYIKIFIMIVGGLIGLRIVF
TVLSIVNRVRQGYSPLSFQTRLPAQRG-PDRPEGIEEEGGERDRDRSGPLVDGFFAIIWV
DLRNLCLFIYHRLRDLLLIVTRIVELLGR-RGWEILKYWWNLLQYWRQELKNSAISLLNA
TAIAVAEGTDRVIEVVQRIGRGILHIPVRIRQGLERALL
>V08_DA_PRAp_2G2.2
MRVRGIRKNYQRLWKWG-TLLSMLLGLLMICNAKEQLWVTVYYGVPVWREANTTLFCASD
ARAYSTEKHNVWATHACVPTDPDPQEVKLGNVTENFNMWKNNMVDQMHEDIISLWDQSLK
PCVEITPLCVTLNCTDYRGNDNSTN------NSNETIVNKEEIKNCSFNITTSLSDKR-Q
ERALFYKYDITPINS-----TTNTSYRLTRCNSSVITQACPKVSFEPIPIHYCAPAGFAI
LKCRQKNFKGTGPCTDVSTVQCTHGIRPVVSTQLLLNGSLAEEEVVIKSESFTDNSKNII
VQLKEAINITCTRPNNNTRKSINI--GPGRAIYATGEIIGDIRQAHCNISEEKWNETLGQ
IVKKLQEQFG-NKSIIFAQPAGGDPETVMHSFNCGGEFFYCNTTRLFNSTWNDSS----T
RSNGTGNNTITLPCRIKQVINMWQKVGKAMYAPPIAGQIRCSSNITGLILTRDGG----S
NNMSNETFRPGGGNMKDNWRSELYKYKVVKIEPLGIAPTKAKRRVVQREKRAIG-IGAVF
LGFLGAAGSTMGAASVTLTVQARQLLFGIVQQQNNLLRAIEAQQHLLQLTVWGIKQLQAR
VLAVERYLKDQQLLGIWGCSGKLICTTAVPWNISWSNKSLEQIWNNMTWMQWEKEINNYT
GLIYTLIEQSQNQQEKNEQELLELDTWASLWNWFDISKWLWYIKIFIMIVGGLIGLRIVF
TVLSIVNRVRQGYSPLSFQTRPPAQRG-PDRPEGIEEEGGERDRDRSGPLVDGFFAIIWV
DLRNLCLFIYHRLRDLLLIVTRIVELLGR-RGWEILKYWWNLLQYWRQELKNSAISLLNA
TAIAVAEGTDRVIEVVQRIGRGILHIPVRIRQGLERALL
>V08DA_POVIp_1H8.6
MRVRGIRKNYQRLWKWG-TLLSMLLGLLMICNAKEQLWVTVYYGVPVWKEANTTLFCASD
ARAYSTEKHNVWATHACVPTDPDPQEVGLGNVTENFNMWKNNMVDQMHEDIISLWDQSLK
PCVEITPLCVTLNCTDYRGNDNSTN------NSNETIVNKEGIKNCSFNITTSLSDKR-Q
ERALFYKYDITPINN-----TTNTSYKLTRCNSSVITQACPKVSFEPIPIHYCAPAGFAI
LKCRQKNFNGTGPCTNVSTVQCTHGIRPVVSTQLLLNGSLAEEEVVIKSENFTDNSKNII
VQLKEAINITCTRPNNNTRKSINI--GPGRAIYATGEIIGDIRQAHCNISEEKWNETLGQ
IVEKLQKQFG-NKSIIFAQPSGGDPETIMHSFNCGGEFFYCNTTQLFNSTWNDSS----T
RSNGTGNNTITLPCRIKQVINMWQKVGKAMYAPPIAGQIRCSSNITGLILTRDGG-----
SNMSNETFRPGGGNMKDNWRSELYKYKVVKIEPLGIAPTKAKRRVVQREKRAIG-IGAVF
LGFLGAAGSTMGAASVTLTVQARQLLFGIVQQQNNLLRAIEAQQHLLQLTVWGIKQLQAR
VLAVERYLKDQQLLGIWGCSGKLICTTAVPWNISWSDKSLEQIWNNMTWMQWEKEINNYT
GLIYTLIEQSQNQQEKNEQELLELDTWASLWNWFDISKWLWYIKIFIMIVGGLIGLRIVF
TVLSIVNRVRQGYSPLSFQTRPPAQRG-PDRPEGIEEEGGERDRDRSGPLVDGFFAIIWV
DLRNLCLFIYHRLRDLLLIVTRIVELLGR-RGWEILKYWWNLLQYWRQELKNSAISLLNA
TAIAVAEGTDRVIEVVQRIGRGILHIPVRIRQGLERALL
>V08_DA_PRAp_2G9.7
MRVRGIRKNYQRLWKWG-TLLSMLLGLLMICNAKEQLWVTVYYGVPVWEEANTTLFCASD
ARAYSTEKHNVWATHACVPTDPDPQEVGLGNVTENFNMWKNNMVDQMHEDIISLWDQSLK
PCVEITPLCVTLNCTDYRGNDNSTN------NSNETIVNKEEIKNCSFNITTSLSDKV-Q
ERALFYKYDITPINS-----TTNTSYKLTRCNSSVITQACPKVSFEPIPIHYCAPAGFAI
LKCRQKNFNGTGPCTEVSTVQCTHGIRPVVSTQLLLNGSLAEGEVVIKSENFTDNSKNII
VQLKEAINITCTRPNNNTRKSINI--GPGRAIYATGEIIGDIRQAHCNISEEKWNETLGQ
IVEKLQEQFG-NKSIIFTQPSGGDPEIVMHSFNCGGEFFYCNTTRLFNSTWNDSS----T
RSNGTGNNTITLPCRIKQVINMWQKVGKAMYAPPIAGQIRCSSNITGLILTRDGG----S
NNMSNETFRPGGGNMKDNWRSELYKYKVVKIEPLGIAPTKAKRRVVQREKRAIG-IGAVF
LGFLGAAGSTMGAASVTLTVQARQLLFGIVQQQNNLLRAIEAQQHLLQLTVWGIKQLQAR
VLAVERYLKDQQLLGIWGCSGKLICTTAVPWNASWSNKSLEQIWNNMTWMQWEKEINNYT
GLIYTLIEQSQNQQEKNEQELLELDTWASLWNWFDISKWLWYIKIFIMIVGGLIGLRIVF
TVLSIVNRVRQGYSPLSFQTRPPAQRG-PDRPEGIEEEGGERDRDRSGPLVDGFFAIIWV
DLRNLCLFIYHRLRDLLLIVTRIVELLGR-RGWEILKYWWNLLQYWRQELKNSAISLLNA
TAIAVAEGTDRVIEVVQRIGRGILHIPVRIRQGLERALL
>V08DA_POVIp_1E4.1
MRVRGIRKNYQRLWKWG-TLLSMLLGLLMICNAKEQLWVTVYYGVPVWKEASTTLFCASD
ARAYSTEKHNVWATHACVPTDPDPQEVGLGNVTENFNMWKNNMVDQMHEDIISLWDQSLK
PCVEITPLCVTLNCTDYRGNDNSTN------NSNETIANKEEIKNCSFNITTSLSDKV-Q
ERALFYMYDITPINN-----TTNTSYKLTRCNSSVITQACPKVSFEPIPIHYCAPAGFAI
LKCRQKNFNGTGPCTNVSTVQCTHGIRPVVSTQLLLNGSLAEEEVVIKSENITDNSKNII

VQLKEAINITCTRPNNNTRKSINI--GPGRAIYATGEIIGDIRQAHCNISEEKWNETLRQ
IVEKLQKQFG-NKSIIFAQPAGGDPETVMHSFNCGGEFFYCNTTRLFNSTWSTWNDS-ST
LSNGTGNNTITLPCRIKQVINMWQKVGKAMYAPPIAGQIRCSSNITGLILTRDGG----S
NNMSNETFRPGGGNMKDNWRSELYKYKVVKIEPLGIAPTKAKRRVVQREKRAIG-IGAVF
LGFLGAAGSTMGAASVTLTVQARQLLFGIVQQQNNLLRAIEAQQHLLQLTVWGIKQLQAR
VLAVERYLKDQQLMGIWGCSGKLICTTAVPWNASWSNKSLQQIWDNMTWMQWEKEINNYT
GLIYTLIEQSQNQQEKNEQELLELDTWASLWNWFDISKWLWYIKIFIMIVGGLIGLRIVF
TVLSIVNRVRQGYSPLSFQTRPPAQRG-PDRPEGIEEEGGERDRDRSGPLVDGFFAIIWV
DLRNLCLFIYHRLRDLLLIVTRIVELLGR-RGWEILKYWWNLLQYWRQELKNSAISLLNA
TAIAVAEGTDRVIEVVQRIGRGILHIPVRIRQGLERALL
>V08DA_POVIp_2C7.1
MRVRGIRKNYQRLWKWG-TLLSMLLGLLMICNAKEQLWVTVYYGVPVWKEANTTLFCASD
ARAYSTEKHNVWATHACVPTDPDPQEVGLGNVTENFNMWKNNMVDQMHEDIISLWDQSLK
PCVEITPLCVTLNCTDYKGNDNSTN------NSNETIVNKEEIKNCSFNITTSLSDKV-Q
ERALFYMYDITPINN-----TTNTSYRLTRCNSSVITQACPKVSFEPIPIHYCAPAGFAI
LKCRQKNFNGTGPCTNVSTVQCTHGIRPVVSTQLLLNGSLAEEEVVIKSENFTDNSKNII
VQLKEAINITCTRPNNNTRKSINI--GPGRAIYATGEIIGDIRQAHCNISEGKWNKTLGQ
IVEKLQKQFG-NKSIIFTQPSGGDPEIVMHSFNCGGEFFYCNTTRLFNSTWNDSS----T
RSNGTGNNTITLPCRIKQVINMWQKVGKAMYAPPIAGQIRCSSNITGLILTRDGG----S
NNMSNETFRPGGGNMKDNWRSELYKYKVVKIEPLGIAPTKAKRRVVQREKRAIG-IGAVF
LGFLGAAGSTMGAASVTLTVQARQLLFGIVQQQNNLLRAIEAQQHLLQLTVWGIKQLQAR
VLAVERYLKDQQLLGIWGCSGKLICTTAVPWNTSWSDKSLEQIWNNMTWMQWEKEINNYT
GLIYTLIEQSQNQQEKNEQELLELDTWASLWNWFDISKWLWYIKIFIMIVGGLIGLRIVF
TVLSIVNRVRQGYSPLSFQTRPPAQRG-PDRPEGIEEEGGERDRDRSGPLVDGFFAIIWV
DLRNLCLFIYHRLRDLLLIVTRIVELLGR-RGWEILKYWWNLLQYWRQELKNSAISLLNA
TAIAVAEGTDRVIEVVQRIGRGILHIPVRIRQGLERALL
>V08_DA_PRAp_2E12.5
MRVRGIRKNYQRLWKWG-TLLSMLLGLLMICNAKEQLWVTVYYGVPVWKEANTTLFCASD
ARAYSTEKHNVWATHACVPTDPDPQEVGLGNVTENFNMWKNNMVDQMHEDIISLWDQSLK
PCVEITPLCVTLNCTDYRGNDNSTN------NSNETIVNKEEIKNCSFNITTSLSDKV-Q
ERALFYMYDITPINN-----TTNTSYKLTRCNSSVITQACPKVSFEPIPIHYCAPAGFAI
LKCRQKNFNGTGPCTDVSTVQCTHGIRPVVSTQLLLNGSLAEEEVVIKSENFTDNSKNII
VQLKEAINITCTRPNNNTRKSINI--GPGRAIYATGEIIGDIRQAHCNISEEKWNKTLGQ
IVKKLQKQFG-NKSIIFAQPSGGDPEIVMHSFNCGGEFFYCNTTRLFNSTWSTWNDS-ST
GSNGTGNNTITLPCRIKQVINMWQKVGKAMYAPPIAGQIRCSSNITGLILTRDGG----S
NNMSNETFRPGGGNMKDNWRSELYKYKVVKIEPLGIAPTKAKRRVVQREKRAIG-IGAVF
LGFLGAAGSTMGAASVTLTVQARQLLFGIVQQQNNLLRAIEAQQHLLQLTVWGIKQLQAR
VLAVERYLKDQQLLGIWGCSGKLICTTAVPWNTSWSNKSLQQIWDNMTWMQWEKEINNYT
GLIYTLIEQSQNQQEKNEQELLELDTWASLWNWFDISKWLWYIKIFIMIVGGLIGLRIVF
TVLSIVNRVRQGYSPLSFQTRPPAQRG-PDRPEGIEEEGGERDRDRSGPLVDGFFAIIWV
DLRNLCLFIYHRLRDLLLIVTRIVELLGR-RGWEILKYWWNLLQYWRQELKNSAISLLNA
TAIAVAEGTDRVIEVVQRIGRGILHIPVRIRQGLERALL
>V08DA_POVIp_2D12.A.3
MRVRGIRKNYQRWWKWG-TLLSMLLGLLMICNAKEQLWVTVYYGVPVWKEASTTLFCASD
ARAYSTEKHNVWATHACVPTDPDPQEVGLGNVTENFNMWKNNMVDQMHEDIISLWDQSLK
PCVEITPLCVTLNCTDYRGND----------NSNETIVNKEEIKNCSFNITTSLSDKV-Q
ERALFYMYDITPINN-----TTNTSYKLTRCNSSVITQACPKVSFEPIPIHYCAPAGFAI
LKCRQKNFNGTGPCTDVSTVQCTHGIRPVVSTQLLLNGSLAEGEVVIKSENFTDNSKNII
VQLKEAINITCTRPNNNTRKSINI--GPGRAIYATGEIIGDIRQAHCNISEEKWNKTLGQ
IVEKLQKQFG-NKSIIFAQPSGGDPETVMHSFNCGGEFFYCNTTRLFNSTWNDSS----T
LSNGTGNNTITLPCRIKQVINMWQKVGKAMYAPPIAGQIRCSSNITGLILTRDGG----S
NNMSNETFRPGGGNMKDNWRSELYKYKVVKIEPLGIAPTKAKRRVVQREKRAIG-IGAVF
LGFLGAAGSTMGAASVTLTVQARQLLFGIVQQQNNLLRAIEAQQHLLQLTVWGIKQLQAR
VLAVERYLKDQQLLGIWGCSGKLICTTAVPWNTSWSNKSLGQIWDNMTWMQWEKEINNYT
GLIYTLIEQSQNQQEKNEQELLELDTWASLWNWFDISKWLWYIKIFIMIVGGLIGLRIVF
TVLSIVNRVRQGYSPLSFQTRLPAQRG-PDRPEGIEEEGGERDRDRSGPLVNGFFAIIWV
DLRNLCLFIYHRLRDLLLIVTRIVELLGR-RGWEILKYWWNLLQYWRQELKNSAISLLNA
TAIAVAEGTDRVIEVVQRIGRGILHIPVRIRQGLERALL
>V08DA_PRAp_2A5.5
MRVRGIRKNYQRLWKWG-TLLSMLLGLLMICNAKEQLWVTVYYGVPVWKEASTTLFCASD
ARAYSTEKHNVWATHACVPTDPDPQEVGLGNVTENFNMWKNNMVDQMHEDIISLWDQSLK
PCVEITPLCVTLNCTDYRGNDNSTN------NSNETIVNKEEIKNCSFNITTSLSDKV-Q
ERALFYMYDITPINN-----TTNTSYKLTRCNSSVITQACPKVSFEPIPIHYCAPAGFAI
LKCRQKNFNGTGPCTDVSTVQCTHGIRPVVSTQLLLNGSLAEGEVVIKSENFTDNSKNII
VQLKEAINITCTRPNNNTRKSINI--GPGRAIYATGEIIGDIRQAHCNISEEKWNKTLEQ
IVEKLQKQFG-NKSIIFAQPSGGDPETVMHSFNCGGEFFYCNTTRLFNSTWNDSS----T
LSNGTGNNTITLPCRIKQVINMWQKVGKAMYAPPIAGQIRCSSNITGLILTRDGG----S
NNMSNETFRPGGGNMKDNWRSELYKYKVVKIEPLGIAPTKAKRRVVQREKRAIG-IGAVF
LGFLGAAGSTMGAASVTLTVQARQLLFGIVQQQNNLLRAIEAQQHLLQLTVWGIKQLQAR
VLAVERYLKDQQLLGIWGCSGKLICTTAVPWNTSWSNKSLEQIWDNMTWMQWEKEINNYT
GLIYTLIEQSQNQQEKNEQELLELDTWASLWNWFDISKWLWYIKIFIMIVGGLIGLRIVF
TVLSIVNRVRQGYSPLSFQTRLPAQRG-PDRPEGIEEEGGERDRDRSGPLVDGFFAIIWV
DLRNLCLFIYHRLRDLLLIVTRIVELLGR-RGWEILKYWWNLLQYWRQELKNSAISLLNA

TAIAVAEGTDRVIEVVQRIGRGILHIPVRIRQGLERALL
>V06_MO_POVIp_2D8.7
MKVKGIRRNYQLLWRW----GIMLLGILRICNATEELWVTVYYGVPVWKEANTTLFCASD
AKTYDTEAHNVWATHACVPTDPNPQEIVLGNVTENFNMWKNDMVEQMHEDIISLWDQSLK
PCVKLTPLCVKLNCTDASTNTTNSTSTTNSTSGGEERMEKEEIKNCSFKITTSTREKV-K
GSAFFYKTDVVSIAK------DNTSYKLIHCNTSVITQACPKVSFEPIPIHYCAPAGFAI
LKCNNKTFNGTGPCKNVSTVLCTHGIKPVVSTQLLLNGSLAEGEVIIRSENFTNNAKTII
VHLNESVVINCTRPNNNTRRSINM--GPGRAFYAT-GIIGDIRQAYCNISQGKWNYTLKQ
IVKKLREQFGENKTIVFNQSSGGDPEIVTHSFNCGGEFFYCNTTKLFNSTWNTSA----W
HGYQEKNDTITLPCKIRQIINMWQEVGKAMYAPPIAGVIRCSSNITGLLLTRDGG----N
ESEGNEIFRPGGGDMRDNWRSELYKYKVVKIEPLGIAPTKAKRRVVQREKRAVG-IGALF
LGFLGAAGSTMGAASITLTGQARLLLSGIVQQQNNLLRAIEAQQHLLQLTVWGIKQLQAR
ILAVERYLKDQQLLGIWGCSGKLICTTAVPWNTSWSNKSMDNIWNNMTWMEWEREIDNYT
GIIYNLLEDSQYQQEKNEKELLELDKWASLWNWFSITNWLWYIKIFIMIVGGLVGLRIVF
AAFSIINRVRQGYSPLSFQTRFPAPRG-PDRPEGIEEEGGERDRDRSGRLVSGFLPLIWD
DLRNLCLFSYHRLRDLLLIVTRIVELLGR-RGWEALKYWWNLLQYWGQELKNSAVSLLNA
ATIAVAEGTDRIIEVLQGTFRGILHIPTRIRQGLERALL
>V06MO_PRAp_1G3.2_b
MKVKGIRRNYQLLWRW----GIMLLGILMICNATEKLWVTVYYGVPVWKEANTTLFCASD
AKAYDTEVHNVWATHACVPTDPNPQELLLENVTENFNMWKNDMVEQMHEDIISLWDQSLK
PCVKLTPLCVKLNCTDANTSTTNSTSTTNSTSGGEERMEKEEIKNCSFNITTSTQEKV-K
GSAFFYKIDIVPIGN----DTTNTSYKLLHCNTSVITQACPKVSFEPIPIHYCAPAGFAI
LKCNNEKFNGTGPCKNVSTVLCTHGIKPVVSTQLLLNGSLAEGEVMIRSENFTNNAKTII
VHLNESVVINCTRPNNNTRKSINM--GPGRAFYAT-GIIGDIRQAHCNISRTAWNNTLEK
IVKKLREQFGKNKTIVFNQSSGGDPEIVTHSFNCGGEFFYCNTTKLFNSTWNTSM----W
HGSNEQEENVTIPCKIRQIINMWQEVGKAMYAPPIAGVIRCSSEITGLLLTRDGG--NES
ESEGKEIFRPGGGDMRDNWRSELYKYKVVKIEPLGIAPTKAKRRVVQREKRAVG-IGALF
LGFLGAAGSTMGAASIVLTGQARLLLSGIVQQQNNLLRAIEAQQHLLQLTVWGIKQLQAR
VLAVERYLKDQQLLGIWGCSGKLICTTAVPWNASWSNKSQNEIWNNMTWMEWEREIDNYT
GIIYNLLEDSQYQQEKNEKELLELDKWASLWNWFSITNWLWYIRIFIMIVGGLVGLRIVF
AVFSVINKVRQGYSPLSFQTRFPAPRG-PDRPEGIEEEGGERDRDRSGRLVNGFLPLIWD
DLRSLCLFSYRRLRDLLLIATRIVELLGR-RGWEALKYWWNLLQYWGQELKNSAVSLLNA
AAIAVAEGTDRIIEVLQRIFRAILHIPTRIRQGLERALL
>V06_MO_POVIp_1F2.7
MKVKGIRRNYQLLWRW----GIMLLGILRICNATEELWVTVYYGVPVWKEANTTLFCASD
AKAYDKEVHNVWATHACVPTDPTPQEIVLGNVTENFNMWKNDMVEQMHEDIISLWDQSLK
PCVKLTPLCVKLNCTDASTSTTNSTSTTNSTSGGEERMEKEEIKNCSFNITTSTQEKV-K
GSAFFYKIDIVPIGN----DTTNTSYRLIHCNTSVITQACPKVSFEPIPIHYCAPAGFAI
LKCNNETFNGTGPCTNVSTVICTHGIKPVVSTQLLLNGSLAEGEVMIRSENFTNNAKTII
VHLNESVVINCTRPNNNTRKSINM--GPGRAFYAT-GIIGDIRQAHCNISRTAWNNTLKK
IVEKLREQFGKNKTIVFNQSSGGDPEIVTHSFNCGGEFFYCNTTKLFNSTWNTSI----E
HGSKDQEGNITLPCKIRQIINMWQEVGKAMYAPPIAGVIRCSSDITGLLLTRDGG--NES
ESEGKEIFRPGGGDMRDNWRSELYKYKVVKIEPLGIAPTKAKRRVVQREKRAVG-IGALF
LGFLGAAGSTMGAASIMLTGQTRQLLSGIVQQQNNLLRAIEAQQHLLQLTVWGIKQLQAR
VLAVERYLKDQQLLGIWGCSGKLICTTDVPWNTSWSNKTLDKIWNNMTWMEWEREIDNYT
GIIYNLLEDSQYQQEKNEKELLELDKWTSLWTWFSITNWLWYIRIFIMIVGGLVGLRIVF
AVISIINKVRQGYSPLSFQTRFPAPRGRPDRPEGIEEEGGERDRDRSGRLVNGFLPLIWD
DLRSLCLFSYRRLRDLLLIVTRIVELLGR-RGWEALKYWWNLLQYWGQELKNSAVSLLNA
AAIAVAEGTDRIIEVLQRIFRAILHIPTRIRQGLERALL
>V06_MO_PRAp_1E2.6
MKVKGIRRNYQLLWKW----GIMLLGILMICNATEKLWVTVYYGVPVWKEANTTLFCASD
ARAYDKEVHNVWATHACVPTDPNPQEVVLGNVTENFNMWKNDMVEQMHEDIISLWDQSLK
PCVKLTPLCVKLNCTNASTNTTNSTSTTNSTSDDKERMEKEEIKNCSFNITTSTQEKV-K
GSAFFYKIDIVPMGN----DTTNTSYRLIHCNTSVITQACPKVSFEPIPIHYCAPAGFAI
LKCNNETFNGTGPCKNVSTVLCTHGIKPVVSTQLLLNGSLAEGEVMIRSENFTNNAKTII
VHLNEAVVINCTRPNNNTRRSINM--GPGRAFYAT-GIIGDIRQAHCNISRTAWNDTLKK
IVKKLREQFG-NKTIVFNQSSGGDPEIVTHSFNCGGEFFYCNTTKLFSSTWNTST----W
HESNDQGENITLPCKIRQIINMWQEVGKAMYAPPIAGVIRCSSNITGLLLTRDGG----N
GSEGKEIFRPGGGDMRDNWRSELYKYKVVKIEPLGIAPTKAKRRVVQREKRAVG-IGALF
LGFLGAAGSTMGAASIMLTGQTRLLLSGIVQQQNNLLRAIEAQQHLLQLTVWGIKQLQAR
VLAVERYLKDQQLLGIWGCSGKLICTTDVPWDTNWSNKTLDKIWNNMTWMEWEREIDNYT
GIIYNLLEESQYQQEKNEKELLELDKWASLWNWFSITNWLWYIRIFIMIVGGLVGLRIVF
TVLSIINRVRQGYSPLSFQTRFPAPRGRPDRPEEIEEEGGERDRDRSGRLVNGFLPLIWD
DLRSLCLFSYRRLRDLILIVTRIVELLGR-RGWEALKYWWNLLQYWGQELKNSAVSLLNA
AAIAVAEGTDRIIEVLQRIFRAILHIPTRIRQGLERALL
>V04EB_PRAp_1H5.6
MKIKETRKNYQHFWKW----GTMLLGMLLICSAAEQLWVTVYYGVPVWKEATTTLFCASD
AKAYDTEAHNVWATHACVPTDPNPQEVVLGNVTENFNAWKNNMVEQMHEDVISLWDQSLK
PCVKLTPLCVTLNCTDWKNPTNNTNTT-DTNATTVNREELGEIKNCSFNITTSMRDKMQK
AYAPFYKLDVEPIDN------NNTSYRLISCNTSVITQACPKMSFEPIPIHYCAPAGFAI
LKCNNKTFNGKGPCTNVSTVQCTHGIKPVVSTQLLLNGSLAEKEVVIRSENFTDNAKVIT
VHLNDSVGINCTRPNNNTRKSIQI--GPGRAFFETGGIIGNIRQAHCNISQKQWNTTLGQ
IVVKLREQYGSNKTIVFNQSSGGDPEIVMHSFNCGGEFFYCNTTPLFNSTWNGTNG--TW

KGTNGTNGTITIQCRIKQFINMWQGVGKAMYAPPISGIIRCSSNITGLLLTRDGG----N
ETSGTEIFRPAGGNMKDNWRSELYKYKVVKIEPLGVAPTKAKRRVVQREKRAVGTIGAMF
LGFLGAAGSTMGAASMTLTVQARLLLSGIVQQQNNLLRAIEAQQHLLQLTVWGIKQLQAR
VLAVERFLRDQQLLGIWGCSGKLICTTTVPWNASWSNKSLDMIWHNMTWMQWEREIDNYT
QLIYTLIEESQNQQEKNEQELLELDKWASLWNWFSITQWLWYIKISIMIVAGLVGLRIVF
AVLSIVNRVRQGYSPLSFQTRLPTPRG-PDRPEETEEEGGDRDRDRSVRLVNGFLALIWD
DLRSLCLFSYHHLRDLLLIAARIVELLGR-RGWEALKYWWNLLQYWIQELKNSAVSLLNA
TAIAVAEGTDRIIEVVQRACR------------ANRA--
>V04EB_POVIp_2A8.4
MKIKETRKNYQHFWKW----GTMLLGMLLICSAAEQLWVTVYYGVPVWKEATTTLFCASD
AKAYDTEAHNVWATHACVPTDPNPQEVVLGNVTENFNAWKNNMVEQMHEDVISLWDQSLK
PCVKLTPLCVTLNCTDWKNPTNNTNTT-DTNTTIVNREELGEIKNCSFNITTSMRDKMQK
AYAPFYKLDVEPIDN------NNTSYRLISCNTSVITQACPKMSFEPIPIHYCAPAGFAI
LKCNNKTFNGKGPCTNVSTVQCTHGIKPVVSTQLLLNGSLAEKEVVIRSENFTDNAKVIT
VHLNDSVGINCTRPNNNTRKSIQI--GPGRAFFETGGIIGNIRQAHCNISQKHWNTTLGQ
IVVKLREQYGSNKTIVFNQSSGGDPEIVMHSFNCGGEFFYCNTTPLFNSTWNGTNG--TW
KGTNGTNGTITIQCRIKQFINMWQGVGKAMYAPPISGIIRCSSNITGLLLTRDGG----N
ETSGTEIFRPAGGNMKDNWRSELYKYKVVKIEPVGVAPTKAKRRVVQREKRAVGTIGAMF
LGFLGAAGSTMGAASMTLTVQARLLLSGIVQQQNNLLRAIEAQQHLLQLTVWGIKQLQAR
VLAVERFLRDQQLLGIWGCSGKLICTTTVPWNASWSNKSLDMIWHNMTWMQWEREIDNYT
QLIYTLIEESQNQQEKNEQELLELDKWASLWNWFSITQWLWYIKIFIMIVAGLVGLRIVF
AVLSIVNRVRQGYSPLSFQTRLPTPRG-PDRPEETEEEGGDRDRDRSVRLVNGFLALIWD
DLRSLCLFSYHHLRDLLLIAARIVELLGR-RGWEALKYWWNLLQYWIQELKNSAVSLLNA
TAIAVAEGTDRIIEVVQRACR------------ANRA--
>V04EB_POVIp_1D1.1
MKIKETRKNYQHFWKW----GTMLLGMLLICSAAEQLWVTVYYGVPVWKEATTTLFCASD
AKAYDTEAHNVWATHACVPTDPNPQEVVLGNVTENFNAWKNNMVEQMHEDVISLWDQSLK
PCVKLTPLCVTLNCTDWKNPTNNTNTT-DTNATTVNREELGEIKNCSFNITTSMRDKMQK
AYAPFYKLDVEPIDN------NNTSYRLISCNTSVITQACPKMSFEPIPIHYCAPAGFAI
LKCNNKTFNGKGPCTNVSTVQCTHGIKPVVSTQLLLNGSLAEKEVVIRSENFTDNAKVIT
VHLNDSVGINCTRPNNNTRKSIQI--GPGRAFFETGGIIGNIRQAHCNISQKQWNTTLGQ
IVVKLREQYGSNKTIVFNQSSGGDPEIVMHSFNCGGEFFYCNTTPLFNSTWNGTNG--TW
KGTNGTNGTITIQCRIKQFINMWQGVGKAMYAPPISGIIRCSSYITGLLLTRDGG----N
ETSGTEIFRPAGGNMKDNWRSELYKYKVVKIEPLGVAPTKAKRRVVQREKRAVGTIGAMS
LGFLGAAGSTMGAASMTLTVQARLLLSGIVQQQNNLLRAIEAQQHLLQLTVWGIKQLQAR
VLAVERFLRDQQLLGIWGCSGKLICTTTVPWNASWSNKSLDMIWHNMTWMQWEREIDNYT
QLIYTLIEESQNQQEKNEQELLELDKWASLWNWFSITQWLWYIKIFIMIVAGLVGLRIVF
AVLSIVNRVRQGYSPLSFQTRLPTPRG-PDRPEETEEEGGDRDRDRSVRLVNGFLALIWD
DLRSLCLFSYHHLRDLLLIAARIVELLGR-RGWEALKYWWNLLQYWIQELKNSAVSLLNA
TAIAVAEGTDRIIEVVQRACR------------ANRA--
>V04EB_POVIp_1F8.7
MKIKETRKNYQHFWKW----GTMLLGMLLICSAAEQLWVTVYYGVPVWKEATTTLFCASD
AKAYDTEAHNVWATHACVPTDPNPQEVVLGNVTENFNAWKNNMVEQMHEDVISLWDQSLK
PCVKLTPLCVTLNCTDWKNPTNNTNTT-DTNATTTNREELGEIKNCSFNITTSMRDKMQK
AYAPFYKLDVEPIDN------NNTSYRLISCNTSVITQACPKMSFEPIPIHYCAPAGFAI
LKCNNKTFNGKGPCTNVSTVQCTHGIKPVVSTQLLLNGSLAEKEVVIRSENFTDNAKVIT
VHLNDSVGINCTRPNNNTRKSIQI--GPGRAFFETGGIIGNIRQAHCNISQKQWNTTLGQ
IVVKLREQYGSNKTIVFNQSSGGDPEIVMHSFNCGGEFFYCNTTPLFNSTWNGTNG--TW
KGTNGTNGTITIQCRIKQFINMWQGVGKAMYAPPISGIIRCSSNITGLLLTRDGG----N
ETSGTEIFRPAGGNMKDNWRSELYKYKVVKIEPLGVAPTKAKRRVVQREKRAVGTIGAMF
LGFLGAAGSTMGAASMTLTVQARLLLSGIVQQQNNLLRAIEAQQHLLQLTVWGIKQLQAR
VLAVERFLRDQQLLGIWGCSGKLICTTTVPWNASWSNKSLDMIWHNMTWMQWEREIDNYT
QLIYTLIEESQNQQEKNEQELLELDKWASLWNWFSITQWLWYIKIFIMIVAGLVGLRIVF
AVLSIVNRVRQGYSPLSFQTRLPTPRG-PDRPEETEEEGGDRDRDRSVRLVNGFLALIWD
DLRSLCLFSYHHLRDLLLIAARIVELLGR-RGWEALKYWWNLLQYWIQELKNSAVSLLNA
TAIAVAEGTDRIIEVVQRACR------------ANRA--
>V04EB_POVIp_1G4.5
MKIKETRKNYQHFWKW----GTMLLGMLLICSAAEQLWVTVYYGVPVWKEATTTLFCASD
AKAYDTEAHNVWATHACVPTDPNPQEVVLGNVTENFNAWKNNMVEQMHEDVISLWDQSLK
PCVKLTPLCVTLNCTDWKNPTNNTNTT-DTNATTTNREELGEIKNCSFNITTSMRDKMQK
AYAPFYKLDVEPIDN------NNTSYRLISCNTSVITQACPKMSFEPIPIHYCAPAGFAI
LKCNNKTFNGKGPCTNVSTVQCTHGIKPVVSTQLLLNGSLAEKEVVIRSENFTDNAKVIT
VHLNDSVGINCTRPNNNTRKSIQI--GPGRAFFETGGIIGNIRQAHCNISQKQWNTTLGQ
IVVKLREQYGSNKTIVFNQSSGGDPEIVMHSFNCGGEFFYCNTTPLFNSTWNGTNG--TW
KGTNGTNGTITIQCRIKQFINMWQGVGKAMYAPPISGIIRCSSNITGLLLTRDGG----N
ETSGTEIFRPAGGNMKDNWRSELYKYKVVKIEPLGVAPTKAKRRVVQREKRAVGTIGAMF
LGFLGAAGSTMGAASMTLTVQARLLLSGIVQQQNNLLRAIEAQQHLLQLTVWGIKQLQAR
VLAVERFLRDQQLLGIWGCSGKLICTTTVPWNASWSNKSLDMIWHNMTWMQWEREIDNYT
QLIYTLIEESQNQQEKNEQELLELDKWASLWNWFSITQWLWYIKIFIMIVAGLVGLRIVF
AVLSIVNRVRQGYSPLSFQTRLPTPRG-PDRPEETEEEGGDRDRDRSVRLVNGFLALIWD
DLRSLCLFSYHHLRDLLLIAARIVELLGR-RGWEALKYWWNLLQYWIQELKNSAVSLLNA
TAIAVAEGTDRIIEVVQRACR------------ANRA--
>V04EB_PRAp_2D9.5

```
MKIKETRKNYQHFWKW----GTMLLGMLLICSAAEQLWVTVYYGVPVWKEATTTLFCASD
AKAYDTEAHNVWATHACVPTDPNPQEVVLGNVTENFNAWKNNMVEQMHEDVISLWDQSLK
PCVKLTPLCVTLNCTDWKNPTNNTNTT-DTNATTVNREELGEIKNCSFNITTSMRDKMQK
AYAPFYKLDVEPIDN------NNTSYRLISCNTSVITQACPKMSFEPIPIHYCAPAGFAI
LKCNNKTFNGKGPCTNVSTVQCTHGIKPVVSTQLLLNGSLAEKEVVIRSENFTDNAKVIT
VHLNDSVGINCTRPNNNTRKSIQI--GPGRAFFETGGIIGNIRQAHCNISQKQWNTTLGQ
IVVKLREQYGSNKTIVFNQSSGGDPEIVMHSFNCGGEFFYCNTTPLFNSTWNGTNG--TW
KGTNGTNGTITIQCRIKQFINMWQGVGKAMYAPPISGIIRCSSNITGLLLTRDGG----N
ETSGTEIFRPAGGNMKDNWRSELYKYKVVKIEPLGVAPTKAKRRVVQREKRAVGTIGAMF
LGFLGAAGSTMGAASMTLTVQARLLLSGIVQQQNNLLRAIEAQQHLLQLTVWGIKQLQAR
VLAVERFLRDQQLLGIWGCSGKLICTTTVPWNASWSNKSLDMIWHNMTWMQWEREIDNYT
QLIYTLIEESQNQQEKNEQELLELDKWASLWNWFSITQWLWYIKTFIMIVAGLVGLRIVF
AVLSIVNRVRQGYSPLSFQTRLPTPRG-PDRPEETEEEGGDRDRDRSVRLVNGFLALIWD
DLRSLCLFSYHHLRDLLLIAARIVELLGR-RGWEALKYWWNLLQYWIQELKNSAVSLLNA
TAIAVAEGTDRIIEVVQRACR------------ANRA--
>V04EB_PRAp_2A10.7
MKIKETRKNYQHFWKW----GTMLLGMLLICSAAEQLWVTVYYGVPVWKEATTTLFCASD
AKAYDTEAHNVWATHACVPTDPNPQEVVLGNVTENFNAWKNNMVEQMHEDVISLWDQSLK
PCVKLTPLCVTLNCTDWKNPTNNTNTT-DTNATTVNREELGEIKNCSFNITTSMRDKMQK
AYAPFYKLDVEPIDN------NNTSYRLISCNTSVITQACPKMSFEPIPIHYCAPAGFAI
LKCNNKTFNGKGPCTNVSTVQCTHGIKPVVSTQLLLNGSLAEKEVVIRSENFTDNAKVIT
VHLNDSVGINCTRPNNNTRKSIQI--GPGRAFFETGGIIGNIRQAHCNISQKQWNTTLGQ
IVVKLREQYGSNKTIVFNQSSGGDPEIVMHSFNCGGEFFYCNTTPLFNSTWNGTNG--TW
KGTNGTNGTITIQCRIKQFINMWQGVGKAMYAPPISGIIRCSSNITGLLLTRDGG----N
ETSGTEIFRPAGGNMKDNWRSELYKYKVVKIEPLGVAPTKAKRRVVQREKRAVGTIGAMF
LGFLGAAGSTMGAASMTLTVQARLLLSGIVQQQNNLLRAIEAQQHLLQLTVWGIKQLQAR
VLAVERFLRDQQLLGIWGCSGKLICTTTVPWNASWSNKSLDMIWHNMTWMQWEREIDNYT
QLIYTLIEESQNQQEKNEQELLELDKWASLWNWFSITQWLWYIKIFIMIVAGLVGLRIVF
AVLSIVNRVRQGYSPLSFQTRLPTPRG-PDRPEETEEEGGDRDRDRSVRLVNGFLALIWD
DLRSLCLFSYHHLRDLLLIAARIVELLGR-RGWEALKYWWNLLQYWIQELKNSAVSLLNA
TAIAVAEGTDRIIEVVQRACR------------ANRA--
>V04EB_PRAp_2C10.1
MKIKETRKNYQHFWKW----GTMLLGMLLICSAAEQLWVTVYYGVPVWKEATTTLFCASD
AKAYDTEAHNVWATHACVPTDPNPQEVVLGNVTENFNAWKNNMVEQMHEDVISLWDQSLK
PCVKLTPLCVTLNCTDWKNPTNNTNTT-DTNATTVNREELGEIKNCSFNITTSMRDKMQK
AYAPFYKLDVEPIDN------NNTSYRLISCNTSVITQACPKMSFEPIPIHYCAPAGFAI
LKCNNKTFNGKGPCTNVSTVQCTHGIKPVVSTQLLLNGSLAEKEVVIRSENFTDNAKVIT
VHLNDSVGINCTRPNNNTRKSIQI--GPGRAFFETGGIIGNIRQAHCNISQKQWNTTLGQ
IVVKLREQYGSNKTIVFNQSSGGDPEIVMHSFNCGGEFFYCNTTPLFNSTWNGTNG--TW
KGTNGTNGTITIQCRIKQFINMWQGVGKAMYAPPISGIIRCSSNITGLLLTRDGG----N
ETSGTEIFRPAGGNMKDNWRSELYKYKVVKIEPLGVAPTKAKRRVVQREKRAVGTIGAMF
LGFLGAAGSTMGAASMTLTVQARLLLSGIVQQQNNLLRAIEAQQHLLQLTVWGIKQLQAR
VLAVERFLRDQQLLGIWGCSGKLICTTTVPWNASWSNKSLDMIWHNMTWMQWEREIDNYT
QLIYTLIEESQNQQEKNEQELLELDKWASLWNWFSITQWLWYIKIFIMIVAGLVGLRIVF
AALSIVNRVRQGYSPLSFQTRLPTPRG-PDRPEETEEEGGDRDRDRSVRLVNGFLALIWD
DLRSLCLFSYHHLRDLLLIAARIVELLGR-RGWEALKYWWNLLQYWIQELKNSAVSLLNA
TAIAVAEGTDRIIEVVQRACR------------ANRA--
>V02_RP_PRAp_2A12.3
MRVREIRRNSRQFWKW----GIMLLGMLMICSAVEQLWVTVYYGVPVWKDANTTLFCASD
AKAYDTEVHNVWATHACVPTDPNPQEVLLENVTENFNMWKNNMVDQMHEDIISLWDQSLK
PCVKLTPLCVTLDCTDLRNATNTTSN---DTSNGGGMPEGGEMKNCSFNITTSVRDKMQK
TYALFYKLDVEPING------DNTSYRLISCNTSVITQACPKVTFEPIPIHYCAPAGFAI
LKCNDKKFNGTGQCRNVSTVQCTHGIKPVVSTQLLLNGSLAEEEVVIRSVNFTNNAKTII
VQLKEAVQINCTRPNNNTRKGIHI--GPGRAFYATGSIIGNIRQAYCNLSSTKWHNTLQQ
IVKKLREQFG-NKTIIFNQSSGGDPEIVMHSFNCGGEFFYCNSTQLFNSTWTNST-----
EGLNTTEDPITLPCRIKQIINLWQEVGKAMYAPPISGIISCSSNITGLLLTRDGG--KNG
SANNTEIFRPEGGDMRDNWRSELYKYKVVKIEPLGIAPTKAKRRVVQREKRAVG-LGAMF
LGFLGAAGSTMGAAAITLTGQARQLLSGIVQQQNNLLRAIEAQQHMLQLTVWGIKQLQAR
VLAVERYLKDQQLLGIWGCSGKLICPTAVPWNTSWSNRSLEKIWDNMTWMEWEREIDNYT
GLIYSLIEESQNQQEKNEQELLELDKWASLWNWFNITQWLWYIKIFIMIVGGLVGLRIVF
AVFSIVNRVRQGYSPLSFQTLLPAPRG-PDRPEGIEEEGGERDRDRSGRLVTGFLALIWD
DLRSLCLFSYHRLRDLLLIVARIVELLGR-RGWEALKYWWNLLKYWSQELKSSAISLLNV
TAIAVAEGTDRIVEVLQRICRAFRNIPRRIRQGLERILI
>V02_POVIp_2D4.2
MRVREIRKSSRQFWKW----GIMLLGMLMICSAVEQLWVTVYYGVPVWKDANTTLFCASD
AKAYDTEVHNVWATHACVPTDPNPQEVILENVTENFNMWKNNMVDQMHEDIISLWDQSLK
PCVKLTPLCVTLDCTDLKNATNTTSN---GTSNGGGMPEGGEIKNCSFNITTSVRDKMQK
TYALFYKLDVEPING------DNTSYRLISCNTSVITQACPKVTFEPIPIHYCAPAGFAI
LKCNDKKFNGTGQCRNVSTVQCTHGIKPVVSTQLLLNGSLAEEEVVIRSVNLTDNAKTII
VQLKEAVQINCTRPNNNTRKGIHI--GPGRAFYATGDIIGNIRQAHCNLSSTKWNNTLKQ
IVKKLREQFG-NKTIIFNQSSGGDPEIVMHTFNCGGEFFYCNSTKLFNSTWNSTE-----
ELNTTEGPPITLPCRIKQIINLWQEVGKAMYAPPISGIISCSSNITGLLLTRDGG----N
GSETNETFRPGGGNMRDNWRSELYKYKVVKIEPLGIAPTKAKRRVVQREKRAVG-LGAMF
```

LGFLGAAGSTMGAAAITLTGQARQLLSGIVQQQNNLLRAIEAQQHMLQLTVWGIKQLQAR
VLAVERYLKDQQLLGIWGCSGKLICPTAVPWNTSWSNRSLEKIWNNMTWMEWEREIDNYT
GLIYSLIEESQNQQEKNEQELLELDKWASLWNWFNITQWLWYIKIFIMIVGGLVGLRIVF
AVFSIVNRVRQGYSPLSFQTLLPAPRG-PDRPEGIEEEGGERDRDRSGRLVTGFLALIWD
DLRSLCLFSYHRLRDLLLIVARIVELLGR-RGWEALKYWWNLLKYWSQELKSSAISLLNV
TAIAVAEGTDRIVEVLQRTCRAFLNIPRRIRQGLERILI
>V02RP_POVIp_1A5.8_2
MRVREIRKSSRQFWKW----GIMLLGMLMICSAVEQLWVTVYYGVPVWKDANTTLFCASD
AKAYDTEVHNVWATHACVPTDPNPQEVILENVTENFNMWKNNMVDQMHEDIISLWDQSLK
PCVKLTPLCVTLDCTDLKNATNTTSN---GTSNGGGMPEGGEMKNCSFNITTSVRDKMQK
TYALFYKLDVEPING------DNTSYRLISCNTSVITQACPKVTFEPIPIHYCAPAGFAI
LKCNDKKFNGTGQCRNVSTVQCTHGIKPVVSTQLLLNGSLAEEEVVIRSVNLTDNAKTII
VQLKEAVQINCTRPNNNTRKGIHI--GPGRAFYATGDIIGNIRQAHCNLSSTKWNNTLKQ
IVKKLREQFG-NKTIIFNQSSGGDPEIVMHTFNCGGEFFYCNSTKLFNSTWNSTE-----
ELNTTEGPPITLPCRIKQIINLWQEVGKAMYAPPISGIISCSSNITGLLLTRDGG----N
GSETNETFRPGGGNMRDNWRSELYKYKVVKIEPLGIAPTKAKRRVVQREKRAVG-LGAMF
LGFLGAAGSTMGAAAITLTGQARQLLSGIVQQQNNLLRAIEAQQHMLQLTVWGIKQLQAR
VLAVERYLKDQQLLGIWGCSGKLICPTAVPWNTSWSNRSLEKIWNNMTWMEWEREIDNYT
GLIYSLIEESQNQQEKNEQELLELDKWASLWNWFNITQWLWYIKIFIMIVGGLVGLRIVF
AVFSIVNRVRQGYSPLSFQTLLPAPRG-PDRPEGIEEEGGERDRNRSGRLVTGFLALIWD
DLRSLCLFSYHRLRDLLLIVARIVELLGR-RGWEALKYWWNLLKYWSQELKSSAISLLNV
TAIAVAEGTDRIVEVLQRTCRAFLNIPRRIRQGLERILI
>V09JF_PRAp-2A11.4
MKAKEIRKNCQRLWRW----GILLLGMLMICSATEKLWVTVYYGVPVWKEANTTLFCASD
AKAYDTEAHNVWATHACVPTDPNPQEVVLANVTENFNMWKNDMVEQMHQDIISLWDQSLK
PCVKLTPLCVTLNCTNLNTNNTNNSSGETNNNANNSSGDYGEIKNCSFNITTPIRNKVQK
EYALFNRLDIVPIDD------DNASYTLISCNTSVITQACPKISFDPIPIHYCAPAGFAI
LKCNNKTFNGTGPCTNVSTVQCTHGIRPVVSTQLLLNGSLAEKETVIRSENFTNNAKTII
VQLNEPIPINCTRPNNNTRKSIHI--APGRAFYATGTIIGNIRQAHCNISNTTWQNALKN
VVAKLREQFG-NKTIVFNQSSGGDPEITMHTFNCGGEFFYCDTTRLFNSTWGFNDT---S
DRANSTNSNITLPCRIKQIINRWQEVGRAMYAPPIRGQIRCSSNITGLLLTRDGGNETEE
NKTNTETFRPGGGNMKDNWRSELYKYKVVKIEPLGVAPTKAKRRVVQREKRAVG-LGAMF
LGFLGAAGSTMGAASITLTVQARQLLSGIVQQQNNLLRAIEAQQHMLQLTVWGIKQLQAR
VLAVERYLKDQQLLGLWGCSGKLICTTAVPWNTSWSNKSQTEIWNNMTWMEWEREINNYT
KVIYTLIEESQNQQEKNEQDLLELDTWAGLWNWFDITNWLWYIKIFIMIVGGLIGLRIVF
TVLSIVNRVRQGYSPLSLQTLFPGPRG-PDRPEGIEEGGGERDRNGSRTLVHGFLALVWV
DLRSLCLFSYHRLRDLLLIVTRTVELLGR-RGWEALKYWWNILQYWSQELRSSAVSLLNA
TAIAVAEGTDRIIEVVQRACRAILHIPRRIRQGAERLLL
>V09JF_POVIp-1D4.2
MKAKEIRKNCQRLWRW----GVLLLGMLMICSATEKLWVTVYYGVPVWKEANTTLFCASD
AKAYDTEAHNVWATHACVPTDPNPQEVVLANVTENFNMWKNDMVEQMHQDIISLWDQSLK
PCVKLTPLCVILNCTNLNTNNTNN-------SSGDYGEIKGEIKNCSFNITTPIRNKVQK
EYALFNRLDIVPIDD------DNASYTLISCNTSVITQACPKISFEPIPIHYCAPAGFAI
LKCNNKTFNGTGPCTNVSTVQCTHGIRPVVSTQLLLNGSLAEKETVIRSENFTNNAKTII
VQLNESIPIDCIRPNNNTRKSIHI--APGRAFYATGTIIGDIRQAYCNISNTTWQNALKK
IVTKLREQFG-NKTIVFNKSSGGDPEITMHTFNCGGEFFYCDTTGLFNSTWGFNDT---S
DWAKSTDSNITLPCRIKQIINRWQEVGRAMYAPPIRGQIRCSSNITGLLLTRDGGNETEE
NKTTTETFRPGGGNMKDNWRSELYKYKVVKIEPLGVAPTQAKRRVVQREKRAVG-LGAMF
LGFLGAAGSTMGAASITLTVQARQLLSGIVQQQNNLLRAIEAQQHMLQLTVWGIKQLQAR
VLAVERYLKDQQLLGLWGCSGKLICTTAVPWNTSWSNKSQKEIWDNMTWMEWEREINNYT
KVIYTLIEESQNQQEKNEQELLALDTWASLWNWFDITKWLWYIKIFIMIVGGLIGLRIVF
TVLSIVNRVRQGYSPLSLQTLFPGPRG-PDRPEGIEEGGGERDRNGSRTLVHGFLALVWV
DLRSLCLFSYHRLRDLLLIVTRTVELLGR-RGWEALKYWWNILQYWSQELRNSAVSLLNA
TAIAVAEGTDRIIEVVQRACRAILHIPRRIRQGAERLLL
>V09F_PRAp-2F9.2
MKAKEIRKNCQRLWRW----GILLLGMLMICSATEKLWVTVYYGVPVWKEANTTLFCASD
AKAYDTEAHNVWATHACVPTDPNPQEVVLANVTENFNMWKNDMVEQMHQDIISLWDQSLK
PCVKLTPLCVTLNCTNLNTNNTNNSSR-ETSNANNSSGDYGEIKNCSFNITTPIRNKVQK
EYALFNRLDIVPIDD------DNASYTLISCNTSVITQACPKISFDPIPIHYCAPAGFAI
LKCNNKTFNGTGPCTNVSTVQCTHGIRPVVSTQLLLNGSLAEKEIIIRSENFTNNAKTII
VQLNESIPINCIRPNNNTRKSIHI--APGRAFYATGTIIGNIRQAHCNISNTTWQNALKK
IVTKLREQFG-NKIIVFNQSSGGDPEITMHTFNCGGEFFYCDTTRLFNSTWEFNDT---S
DQANSTNINITLPCRIKQIINRWQEVGRAMYAPPIRGQIRCSSNITGLLLTRDGGNETEE
NKTNTETFRPGGGNMKDNWRSELYKYKVVKIEPLGVAPTQAKRRVVQREKRAVG-LGAMF
LGFLGAAGSTMGAASITLTVQARQLLSGIVQQQNNLLRAIEAQQHMLQLTVWGIKQLQAR
VLAVERYLKDQQLLGLWGCSGKLICTTAVPWNTSWSNKSQTEIWNNMTWMEWEREINNYT
KVIYTLIEESQNQQEKNEQELLALDTWASLWNWFDITKWLWYIKIFITIVGGLIGLRIVF
TVLSIVNRVRQGYSPLSLQTLFPGPRG-PDRPEGIEEGGGERDRNGSRTLVHGFLALVWV
DLRSLCLFSYHRLRDLLLIVTRTVELLGR-RGWEALKYWWNILQYWSQELKNSAISLLNA
TAIAVAEGTDRIIEVVQRACRAILHIPRRIRQGAERILL
>V03NP_POVIp-2D5.10.2
MRVRGIRKNYQHWWGWS-TMLTMLLGMLMICKAAEQLWVTIYYGVPVWKEATTTLFCASD
AKAYDTEMHNVWATHACVPTDPNPQEVVLENVTENFNMWENNMVEQMHEDIISLWDQSLK

```
PCVKLTPLCVTLNCTNANISSNTSN------IYNSSIYKPGDIKNCSFNITTHIRDKVKK
EYALFYALDVIPIDPTVGNKTTTTDYRLISCNTSVVTQACPKVSFEPIPIHYCAPAGFAI
LKCNDKMFNGTGPCKNVSTIQCTHGIRPVVSTQLLLNGSLAEEDIVIRSKNFTDNAKTII
VQLNKTVTINCTRPSNNTRKSIHI--APGRAFYATGNIIGDIRQAHCNLSRKDWNSTLRL
IANKLREQFG-NKTIVFNSSSGGDPEIVMHSFNCRGEFFYCNTTPLFNSTWYSNN----T
QEGMEGNSIITLQCRIKQIVNMWQEVGKAMYAPPIKGQIRCSSNITGLLLTRDGG----N
TTNRPEVFRPGGGNMKDNWRSELYKYKVVKLEPLGVAPTKAKRRVVQREKRAVG-IGALF
LGFLGAAGSTMGAASMTLTVQARQLLSGIVQQQNNLLMAIEAQQHMLQLTVWGIKQLQAR
VLAVESYLKDQQLLGIWGCSGKLICTTTVPWNTSWSNKTYNEIWDNMTWMQWEKEIDNHT
SLIYTLIEESQNQQEKNELDLLALDKWANLWSWFNITNWLWYIKIFIMIVGGLVGLRIVF
TVLSIVNRVRQGYSPLSFQTHLPARRG-PDRPEEIEEEGGERDRGRSEPLVTGFLTLIWV
DLRSLCLFSYHRLRDLLLIAARIVELLGR-RGWEILKYWWNLLQYWSQELKNSAVSLLNA
TAIAVAEGTDRVIEIVQGIGRAILHIPARIRQGLERVLL
```
